# Synthesis and Photovoltaic Performance of β-Amino-Substituted Porphyrin Derivatives

**DOI:** 10.3390/ijms25115979

**Published:** 2024-05-29

**Authors:** Ana F. R. Cerqueira, Ana Lucia Pinto, Gabriela Malta, Maria G. P. M. S. Neves, A. Jorge Parola, Augusto C. Tomé

**Affiliations:** 1 LAQV-REQUIMTE, Department of Chemistry, University of Aveiro, 3810-193 Aveiro, Portugal; anacerqueira@ua.pt (A.F.R.C.); gneves@ua.pt (M.G.P.M.S.N.); 2 LAQV-REQUIMTE, Department of Chemistry, NOVA School of Science and Technology, FCT NOVA, Universidade NOVA de Lisboa, 2829-516 Caparica, Portugal; al.pinto@alumni.fct.unl.pt (A.L.P.); g.malta@campus.fct.unl.pt (G.M.)

**Keywords:** porphyrins, nucleophilic aromatic substitution, thiols, dye-sensitized solar cells, power conversion efficiency

## Abstract

New β-amino-substituted porphyrin derivatives bearing carboxy groups were synthesized and their performance as sensitizers in dye-sensitized solar cells (DSSC) was evaluated. The new compounds were obtained in good yields (63–74%) through nucleophilic aromatic substitution reactions with 3-sulfanyl- and 4-sulfanylbenzoic acids. Although the electrochemical studies indicated suitable HOMO and LUMO energy levels for use in DSSC, the devices fabricated with these compounds revealed a low power conversion efficiency (PCE) that is primarily due to the low open-circuit voltage (V_oc_) and short-circuit current density (J_sc_) values.

## 1. Introduction

Fossil fuels such as coal, oil, and natural gas are still the main energy source for industrial and social human activities. However, because they are finite and produce unwanted consequences, namely pollution and climate change, it is becoming important to develop technologies based on renewable and non-polluting energy sources. Sunlight is an endless source of clean and free energy and, thus, harvesting solar energy is an obvious approach for dealing with energy problems. Dye-sensitized solar cells (DSSC) have attracted significant interest for the conversion of sunlight into electricity because they are easily fabricated, have low costs, and are environmentally friendly [1,2]. In general, sensitizers used in DSSC are divided into three main groups: ruthenium complexes [3,4,5], metal-free organic dyes [6,7,8,9], and porphyrin/phthalocyanine dyes [10,11,12,13,14].

One of the most successful DSSC described in the literature uses ruthenium-based dyes, with a power conversion efficiency (PCE) of about 12% [15,16] but, unfortunately, ruthenium is not an earth-abundant element and is expensive [17,18,19]. In order to solve this problem, several researchers have been focused on the replacement of ruthenium complexes by organic dyes that can achieve similar (or better) PCE. Porphyrin derivatives are an effective alternative to ruthenium sensitizers, as they exhibit a series of photophysical properties that are appropriate for DSSC, such as broad absorption in the visible region, and favorable cell kinetics for electron injection and charge recombination [20]. That explains why porphyrins have been so extensively investigated as dyes in DSSC during the last decade [11,13,20,21,22]. In 2014, the performance of porphyrin-based DSSC (PCE = 13%) [23] exceeded that of the ruthenium-based DSSC.

Porphyrins may be functionalized at the *meso-* or β-pyrrolic positions with substituents adequate for anchoring to TiO_2_. Typically, porphyrins used in DSSC present *meso*-carboxyphenyl groups or *meso*-carboxyalkynylphenyl groups while β-substituted porphyrins usually have conjugated alkenyl systems bearing a terminal carboxylic acid [24,25].

In this paper, we report the synthesis of porphyrins bearing β-amino substituents containing a terminal benzoic acid unit. Some of the new porphyrins bear fused rings. The photophysical and electrochemical properties of the new compounds and their performance as dyes in DSSC are also discussed.

## 2. Results and Discussion

### 2.1. Synthesis

**Figure 1:**
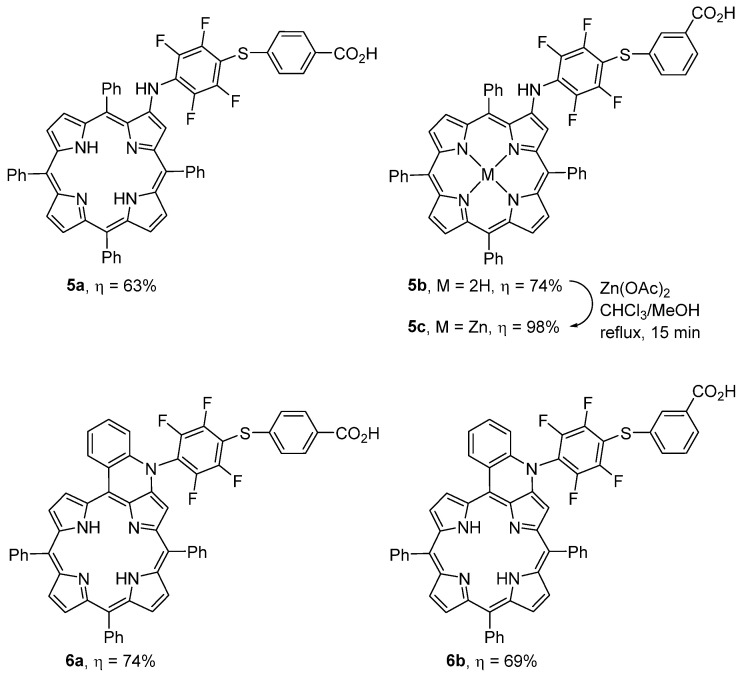
Porphyrin derivatives used in DSSC.

**Scheme 1:**
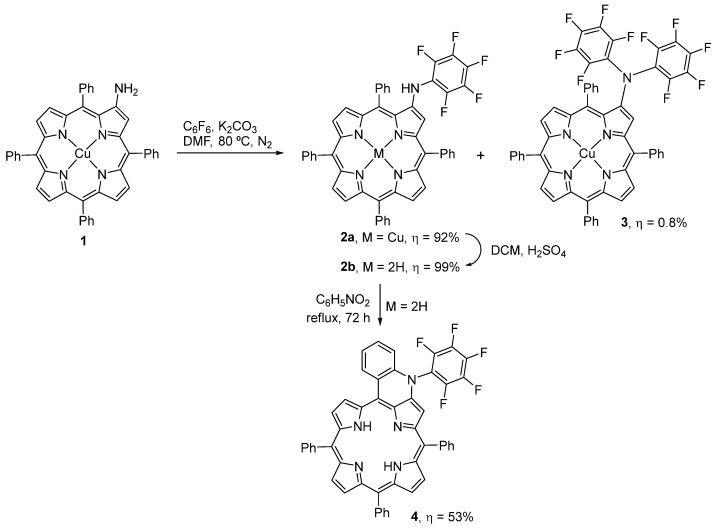
Synthesis of porphyrin derivatives **2**–**4**.

The synthesis of the porphyrin derivatives bearing carboxy groups **5a**–**c** and **6a**,**b** (Figure 1) required the previous preparation of (2-amino-5,10,15,20-tetraphenylporphyrinato)copper(II) (**1**) (Scheme 1). This compound was prepared by nitration of (5,10,15,20-tetraphenylporphyrinato)copper(II) followed by reduction of the 2-nitro group under standard Sn/HCl conditions [26]. The reaction of the 2-aminoporphyrin **1** with hexafluorobenzene was carried out in dimethylformamide (DMF) at 80 °C, for 5 h, and using K_2_CO_3_ as base. The TLC of the reaction mixture revealed the total consumption of the starting porphyrin and the formation of the *N*-pentafluorophenylamino derivative **2a** as the major product (92% yield) accompanied by a minor amount of the bis(pentafluorophenyl)aminoporphyrin **3** (0.8% yield). The formation of compound **3** was not a surprise. In fact, we had already reported the synthesis of a similar porphyrin derivative bearing a bis(pentafluorophenyl)amino group at the *meso* position [27]. The structures of compounds **2a** and **3** were confirmed by their absorption spectra (ESI, Figures S1 and S4) and mass spectra (ESI, Figures S2, S3 and S5) that reveal a peak at *m*/*z* = 857 for **2a** and at *m*/*z* = 1022 for **3**, corresponding to the protonated molecular ion [M + H]^+^ and to the molecular ion M^+^˙ of the proposed structures, respectively.

The demetalation of porphyrin **2a** with a mixture of H_2_SO_4_/CH_2_Cl_2_ afforded the free base **2b** in quantitative yield. The structure of **2b** was confirmed by ^1^H, ^19^F, and ^13^C NMR, UV–Vis and by MS (ESI, Figures S8–S15). Its ^1^H NMR spectrum (ESI, Figure S6) presented the expected signals in the aromatic region due to the resonances of six β-pyrrolic protons (δ 8.60–8.90 ppm), and of the *meso*-phenyl groups plus the β-pyrrolic proton at position three (δ 7.60–8.30 ppm). Additionally, the spectrum showed a singlet at δ 5.95 ppm due to the resonance of one *N*H proton, which is in accordance with the occurrence of a mono-substitution. The ^19^F NMR spectrum (ESI, Figure S7) shows three signals at δ −143.9, −159.3, and −159.6 to −160.4 ppm, with relative intensities 2:2:1, in the form of doublet, triplet, and multiplet, respectively, which can be assigned, respectively, to the *ortho*-, *meta*-, and *para*-fluorine atoms. 

Considering that the extension of the electronic π-system of a porphyrin macrocycle results, in general, in the red-shift of the Q bands (or the appearance of new absorption bands), we decided to synthesize porphyrin derivative **4** bearing a fused ring system. The formation of **4** involved the oxidative cyclization of the 2-aminoporphyrin **2b** following a procedure reported by our group [28]. The desired compound **4** was isolated in 53% yield and its structure was confirmed by ^1^H, ^19^F, and ^13^C NMR, UV–Vis and MS (ESI, Figures S12–S17). The mass spectrum (ESI, Figure S16) showed a peak at *m*/*z* = 794 corresponding to the protonated molecular ion [M + H]^+^. In the ^1^H NMR spectrum (ESI, Figure S12), the absence of the singlet at δ 5.95 ppm, corresponding to the resonance of the *N*H proton observed in **2b**, and the presence of two multiplets at δ 8.15–8.30 ppm, corresponding to the resonances of six *ortho*-Ph-H protons only, are clear evidence that the oxidative cyclization occurred.

Knowing that pentafluorophenyl groups react easily with nucleophiles [27], and particularly with thiols [29], by nucleophilic aromatic substitution reactions, and considering that dyes to be applied in DSSC should contain an anchoring group to bind to the surface of titanium dioxide, we performed the reaction of porphyrins **2b** and **4** with 4-sulfanyl- and 3-sulfanylbenzoic acid. The structures of the resulting porphyrins **5a**,**b** and **6a**,**b** are shown in Figure 1. The nucleophilic aromatic substitution took place in DMF in the presence of K_2_CO_3_ at room temperature. The porphyrin derivatives **5a**,**b** and **6a**,**b** were isolated in good yields (63–74%) and their structures were confirmed by ^1^H, ^19^F, and ^13^C NMR, UV–Vis and MS (ESI, Figures S18–S41). The main evidence that the nucleophilic aromatic substitution occurred was observed in the ^19^F NMR spectra. For example, the spectrum of **5a** showed two main signals, in the form of doublets, integrating for two protons each, at δ −132.1 and −149.0 ppm (ESI, Figure S19).

In order to evaluate the influence of the porphyrin metalation on the efficiency of DSSC devices, it was decided to prepare the Zn(II) complex **5c**. The metalation was carried out by adding zinc(II) acetate to a solution of **5b** in chloroform and methanol. After crystallization from dichloromethane/methanol, compound **5c** was obtained in 98% yield. The structure of the Zn(II) complex **5c** was confirmed by its ^19^F NMR, UV–Vis and MS spectra (ESI, Figures S42–S44). It was not possible to obtain the ^1^H NMR spectrum of this compound due to their very low solubility (probably due to the formation of aggregates or ordered polymeric structures, resulting from intermolecular Zn–amine interactions) [30,31].

### 2.2. Spectroscopic Properties

**Figure 2:**
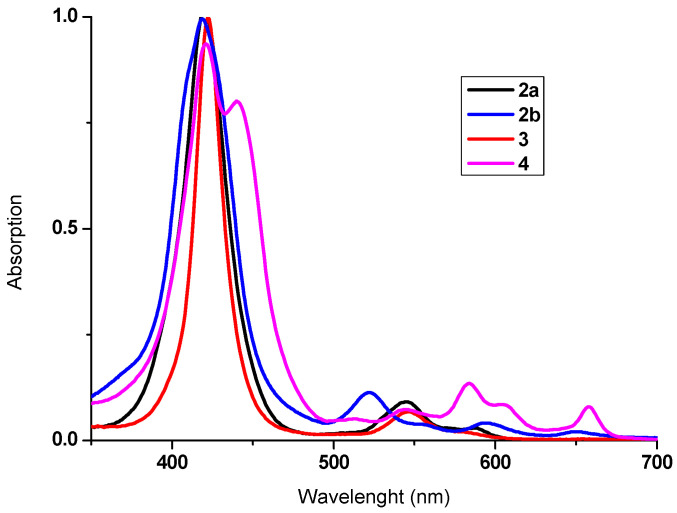
UV–Vis absorption spectra of porphyrin derivatives **2**–**4** (10^−5^–10^−6^ mol L^−1^ in CHCl_3_).

**Figure 3:**
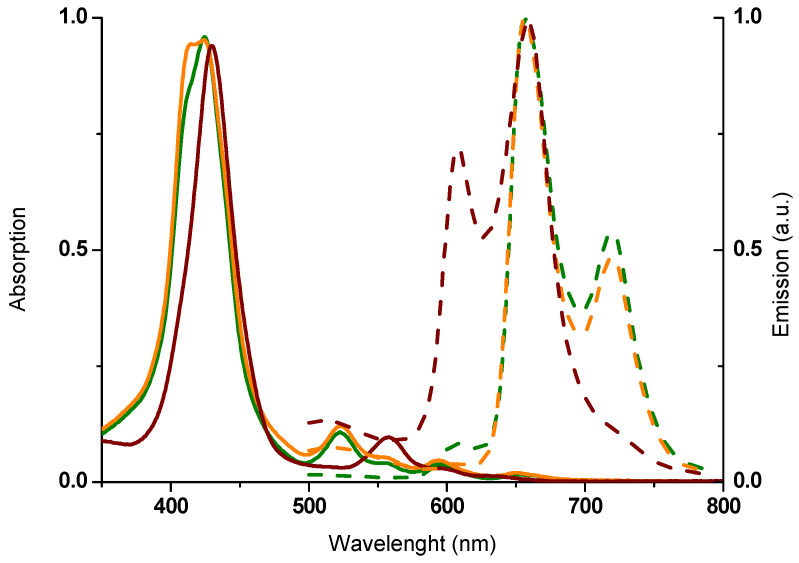
UV–Vis absorption (solid lines) and normalized emission (dotted lines) spectra of porphyrin derivatives **5a** (green lines), **5b** (orange lines), and **5c** (brown gray lines) (10^−5^–10^−6^ mol L^−1^ in CHCl_3_).

**Figure 4:**
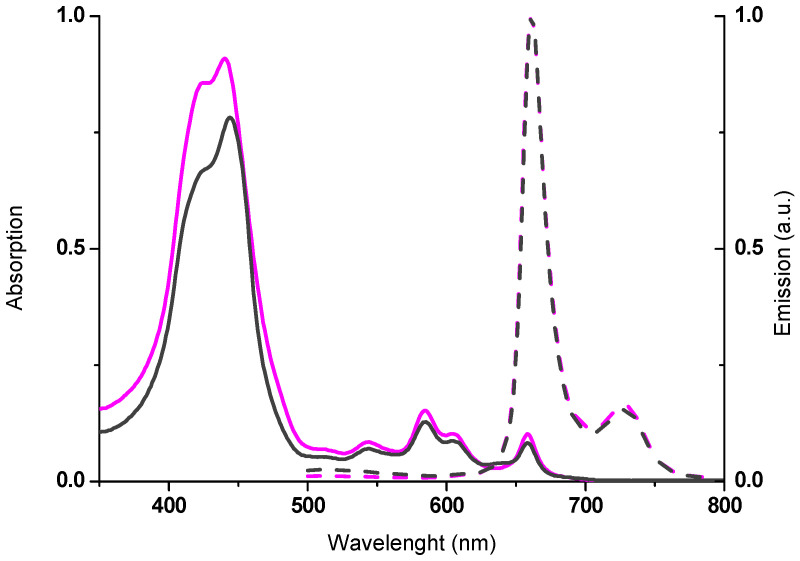
UV–Vis absorption (solid lines) and normalized emission (dotted lines) spectra of porphyrin derivatives **6a** (magenta lines) and **6b** (dark gray lines) (10^−5^–10^−6^ mol L^−1^ in CHCl_3_).

The absorption and emission spectra of porphyrin derivatives **2**–**6** in chloroform are shown in Figure 2, Figure 3 and Figure 4 and summarized in Table 1 for compounds **5a**–**c** and **6a**,**b**. As expected, all free-base porphyrins (**2b**, **4**, **5a**, **5b**, **6a**, **6b**) exhibit absorption spectra composed of an intense Soret band (415–444 nm) and four less intense Q bands (*ca*. 500–658 nm). In turn, metalated derivatives **2a**, **3**, and **5c** show fewer Q bands as a result of a change in symmetry and orbital degeneracy upon complexation [32]. Metalation leads to a higher planarization of the porphyrin, with increased delocalization and red shift of the absorption bands, as observed when comparing **2a** with **2b**, and **5c** with **5a** and **5b**. Leaving behind the synthetic intermediate porphyrins **2**–**4**, a more detailed analysis of the spectroscopic properties of the final dyes **5**–**6** shows no significant difference between the *meta*- and *para*- substitution of the carboxylic acid group in the fluorinated benzene ring. More significant is the rigidification imposed by ring fusion when transitioning from porphyrins **5** to porphyrins **6**, as can be noticed by the different colors of both types of porphyrins (orange-red for **5**; greenish for **6** when adsorbed on TiO_2_). The more conjugated ring-fused porphyrins **6** present red-shifted bands when compared to **5** (16–20 nm in the Soret band, 7–8 nm in the Q4 band), with significant splitting of the Soret band. The emission spectra reflect corresponding red-shifts in the Soret and Q bands. The smaller Stokes shift of porphyrins **6** relative to **5** reflect the higher rigidification in the cyclized porphyrins. In the case of the metalated derivative **5c**, a smaller red-shift could also be expected relative to **5b** due to planarization, but the lowest-energy Q band is too broad for calculating an accurate absorption maximum.

### 2.3. Electrochemical Characterization

**Figure 5:**
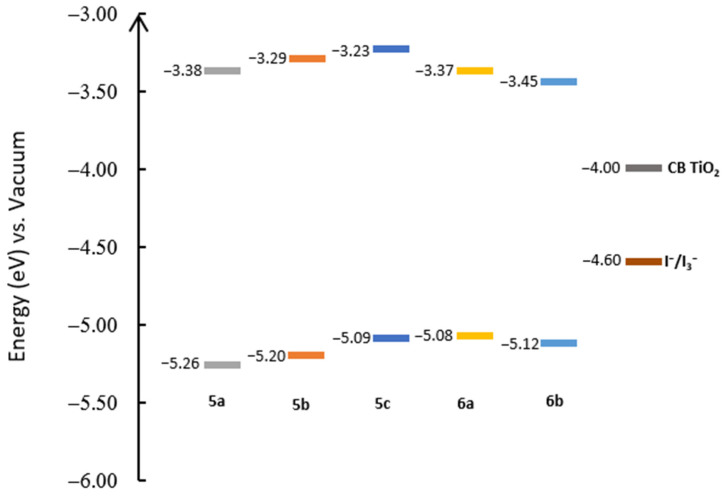
Energy-level diagram of porphyrin derivatives **5a**–**c**, **6a**,**b**, the electrolyte (I^−^/I_3_^−^), and the CB of TiO_2_.

The electrochemical properties of compounds **5** and **6** were studied using differential pulse voltammetry (DPV) aiming to evaluate the suitability of these porphyrins as sensitizers for DSSC. The electrochemical data and the energy-level diagram are summarized in Table 2 and Figure 5, respectively. From the obtained onsets of the oxidation and reduction peaks, the values of the HOMO and LUMO energies were calculated with the following equation: E [eV] = −(E_onset_ (V vs. SCE) + 4.44) [33]. The onset values were considered the intersection points between the tangent lines of the rising current and the baseline current (see Supplementary Materials, Figures S45–S49). Additionally, the HOMO/LUMO potentials of porphyrins **5** and **6** were compared with those of the electrolyte (I^−^/I_3_^−^ redox pair) and the conducting band (CB) of TiO_2_. The highest occupied molecular orbital energy levels, located between −5.26 and −5.08 eV, are more negative than the I^−^/I_3_^−^ redox couple potential (−4.60 eV) [34], which ensures efficient regeneration of the oxidized dye. The lowest unoccupied molecular orbital energy levels, located between −3.45 and −3.23 eV, are higher than the CB of TiO_2_ (−4.0 eV) [34], indicating that electron injection from the excited state of the dyes to the CB of TiO_2_ surface is thermodynamically permitted.

Porphyrins with fused rings (**6a** and **6b**) have lower band gap values due to the extension of the conjugated system, corroborating the observed red-shifts when transitioning from porphyrins **5a** and **5b** to **6a** and **6b**. This rigidification also results in lower oxidation potentials when compared to the non-rigidified analogs, suggesting an increased ability to donate electrons. It was expected that this would lead to an improvement in photocurrent generation when compared with **5a** and **5b**, but no correlation was observed. The Zn(II) complex **5c** has higher HOMO and LUMO levels and a narrower bandgap when compared with **5b**.

### 2.4. Fabrication of DSSC Using the Porphyrin Derivatives ***5a***–***c*** and ***6a**,**b***

**Figure 6:**
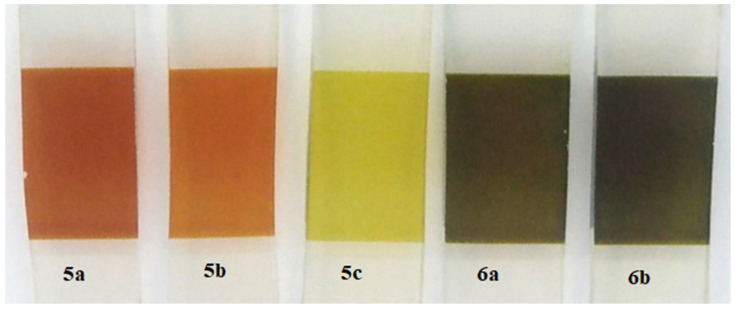
Pictures of the colored films resulting from the adsorption of the 0.5 mM dichloromethane solutions of the porphyrin derivatives **5a**–**c** and **6a**,**b** on TiO_2_ thin films (*ca*. 6 µm).

The preparation of the DSSC devices with each porphyrin **5a**–**c** and **6a**,**b** is detailed in the experimental section. Upon adsorption onto a thin film of TiO_2_ deposited on the surface of an FTO-coated glass slide, all compounds resulted in highly colored and homogeneous films (Figure 6).

**Figure 7:**
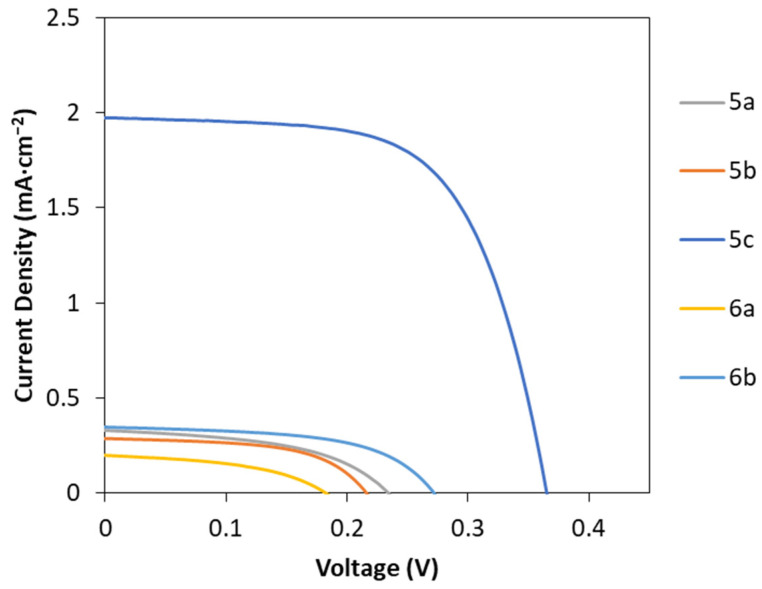
I–V curves for the studied compounds adsorbed from 0.5 mM solutions in dichloromethane measured under 100 mW cm^−2^ simulated AM 1.5G illumination, using 0.8 M LiI and 0.05 M I_2_ in acetonitrile/pentanenitrile (85:15, % *v*/*v*) as electrolyte.

The photovoltaic performance of the resulting DSSC devices was compared with that obtained from the dye N719, which was selected as reference. The results were obtained through I–V measurements under 100 mW cm^−2^ simulated AM1.5G illumination, using 0.8 M LiI and 0.05 M I_2_ in acetonitrile/pentanenitrile (85:15, % *v*/*v*) as electrolyte. The results summarized in Figure 7 and Table 3 show that all porphyrin derivatives exhibit low efficiency with PCE values varying between 0.4% and 10% of the value obtained for N719.

The low performance of these dyes is primarily due to their low open-circuit voltage (V_oc_) and the short-circuit current density (J_sc_) values (Table 3). This outcome can be attributed to the common occurrence of aggregation in this family of compounds and/or to an inefficient electron-injection from the excited molecule to the semiconductor.

A comparison between the DSSC performance using the free-base dyes **5a** and **5b** allows us to conclude that the position of the carboxy group in the terminal benzoic acid unit has no significant effect (Table 2). This is in line with the very similar absorption spectra observed for both dyes, and also the observed similar bandgap. It could be that the position of the carboxy group would lead to a different orientation of the dye on the surface of the anode, as observed in other porphyrin systems [35], but the similar poor efficiency of the DSSC cells of **5a** and **5b** suggests that no such effect is present. Comparing **5b** and **5c**, the presence of Zn(II) in **5c** leads to a noticeable efficiency enhancement with an increase in the PCE from 0.02 to 0.37%. This improvement can be attributed to an increase of approximately 160 mV in V_oc_ (1.8-fold increase) and of 1.6 mA cm^−2^ in the J_sc_ value (7.8-fold increase). Large enhancements in DSSC efficiency upon Zn metalation in porphyrins have been attributed to a mediator role of the metal ion in the injection step [36]. The closed-shell nature of the Zn(II) ion with empty coordination sites can allow for a rapid injection of photoexcited porphyrin electrons into the TiO_2_ conduction band, reflected in the high increase in J_sc_ in **5c** when compared to **5b**.

When comparing the PCE values obtained for the non-cyclized derivatives **5** with those of the corresponding rigidified structures **6**, there is no apparent pattern for the rigidification effect that can be established. For instance, a decrease in the PCE was observed when comparing **5a** with **6a** (0.03% vs. 0.013%), while the opposite was observed for the other pair **5b** and **6b** (0.02% vs. 0.049%).

The overall low efficiencies of the DSSC devices built with these porphyrin derivatives may be associated with the presence of the electronegative fluorine atoms, which might lead to an inefficient electron-injection from the excited molecule to the semiconductor.

### 2.5. Effect of CDCA Addition on the DSSC Performance

To assess if the low efficiency results obtained for the DSSC are due to the formation of aggregates, studies with chenodeoxycholic acid (CDCA) were performed. CDCA is a recognized de-aggregating agent commonly employed in DSSC. The incorporation of CDCA as a co-adsorber in DSSC has been shown to enhance cell performance by influencing the photocurrent through increased charge collection and/or electron injection, as well as by improving open-circuit photovoltage through the suppression of charge recombination [37,38,39,40].

**Figure 8:**
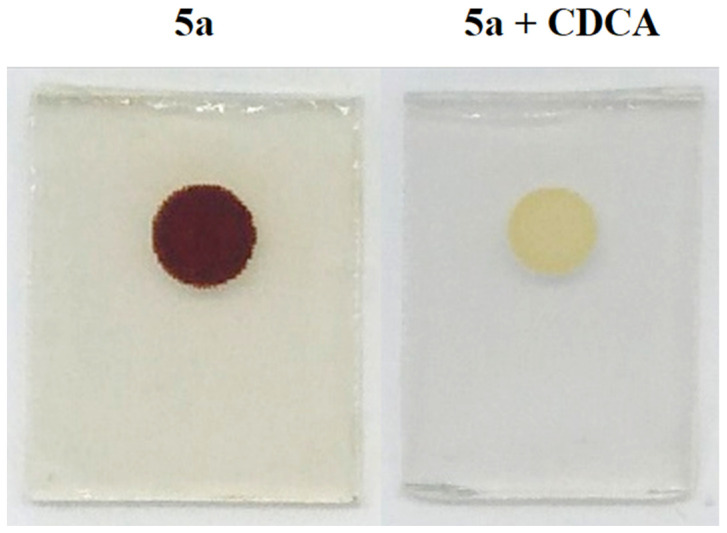
Pictures of the photoanodes resulting from the adsorption of **5a** (from a 0.5 mM dichloromethane solution) and the adsorption of the same compound in the presence of 50 mM of CDCA.

In this study, 50 mM of CDCA was added to the solution of **5a**, as well as a few drops of methanol to facilitate the solubilization of CDCA. The outcome of this operation was a significant decrease in the adsorbed dye (Figure 8), suggesting a direct competition of CDCA for the anchoring sites on the TiO_2_ surface.

**Figure 9:**
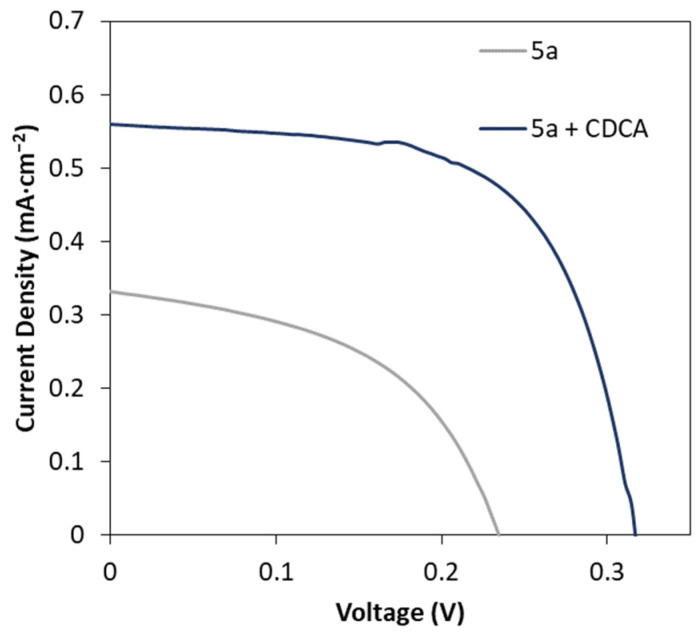
Comparison of I–V curves based on compound **5a** adsorbed from 0.5 mM solutions in dichloromethane and **5a** plus 50 mM CDCA, measured under 100 mW cm^−2^ simulated AM 1.5G illumination, using 0.8 M LiI and 0.05 M I_2_ in acetonitrile/pentanenitrile (85:15, % *v*/*v*) as electrolyte.

The addition of CDCA had a positive impact on the performance of the resulting DSSC, as evident from the outcomes detailed in Table 4 and in Figure 9. The incorporation of CDCA resulted in the improvement in all cell parameters, resulting in a final efficiency of 0.11%. This increment in photocurrent and photovoltage can be attributed to the de-aggregating effect, which will avoid quenching and, thus, facilitate charge injection, as well as to TiO_2_ surface passivation by CDCA molecules, which translates into the clear increase verified in the FF, from 0.41 to 0.63, possibly indicating a decrease in dark current phenomena. The increase in PCE upon the addition of CDCA is, however, limited by the lower adsorption of the dye which led us to not extend this test to the other porphyrins.

## 3. Materials and Methods

### 3.1. Materials

All chemicals were used as provided. Solvents were used as received or distilled and dried using standard procedures according to literature procedures [41]. The 2-amino-5,10,15,20-tetraphenylporphyrinato)copper(II) (**1**) was obtained by nitration of 5,10,15,20-tetraphenylporphyrin (TPP), followed by reduction of the 2-nitro derivative, according to literature procedures [26].

### 3.2. Methods

^1^H and ^13^C NMR spectra were recorded on a Bruker Avance 300 (at 300.13 and 75.47 MHz, respectively) or on a Bruker Avance 500 (at 500 and 125 MHz, respectively) spectrometers (Bruker, Wissembourg, France). ^19^F NMR spectra were obtained on a Bruker Avance 300 at 282 MHz. CDCl_3_ or DMSO-*d_6_* were used as solvents with tetramethylsilane (TMS) as the internal reference. Chemical shifts are expressed in δ (ppm) and the coupling constants (*J*) in hertz (Hz). UV–Vis spectra were recorded on a Shimadzu UV-2501PC spectrophotometer (Shimatzu, Kyoto, Japan) using CHCl_3_ as solvent. λ_max_ values are in nm and log ε values were calculated from molar absorptivity in M^−1^cm^−1^. The emission spectra were recorded on a Horiba Jobin-Yvon Fluoromax 4 spectrofluorimeter (Shimatzu, Kyoto, Japan) using CHCl_3_ as solvent. Mass spectra (MS) were recorded using a Micromass Q-TOF-2TM mass spectrometer (Micromass, Manchester, UK) and CHCl_3_ as solvent. High-resolution mass spectra (HRMS-ESI) were obtained in a Q-Exactive^®^ hybrid quadrupole Orbitrap^®^ mass spectrometer (Thermo Fisher Scientific, Bremen, Germany). The instrument was operated in positive mode, with a spray voltage at 3.0 kV, and interfaced with a HESI II ion source. The analyses were performed through direct infusion of the prepared solutions at a flow rate of 10 μL min^−1^ into the ESI source. Spectra were analyzed using the acquisition software Xcalibur (ver. 4.0, Thermo Scientific, San Jose, CA, USA). Preparative thin layer chromatography was carried out on 20 cm × 20 cm glass plates coated with silica gel (1 mm thick). Column chromatography was performed using silica gel (Merck (Darmstadt, Germany), 35–70 mesh). Analytical TLC was carried out on precoated sheets with silica gel (Merck 60, 0.2 mm thick). 

### 3.3. Synthesis and Characterization

#### 3.3.1. Synthesis of **2a**

A solution of **1** (185 mg, 268 µmol), hexafluorobenzene (2 mL), and potassium carbonate (222 mg, 1.6 mmol, 6 equiv.) in dry DMF (4 mL) was stirred at 80 °C for 5 h under a nitrogen atmosphere. After cooling at room temperature, the reaction mixture was diluted with dichloromethane and washed with water. The organic phase was dried (Na_2_SO_4_) and the solvent was evaporated under reduced pressure. The residue was purified by column chromatography (silica gel) using dichloromethane/hexane (1:1) as the eluent. The fraction with higher R_f_ was identified as the bis(pentafluorophenyl)aminoporphyrin **3** (2.1 mg, 0.8% yield). A second fraction was identified as the porphyrin **2a** (210 mg, 92% yield).

**2a**: UV–Vis (CHCl_3_): λ_max_ (log ε) 420 (4.63), 545 (3.58), 587 (3.08). MS (ESI(+)): *m*/*z* 857.3 [M + H]^+^. HRMS-ESI(+): *m*/*z* calcd. for C_50_H_29_CuF_5_N_5_ [M + H]^+^ 857.1634; found 857.1631.

**3:** UV–Vis (CHCl_3_): λ_max_ (log ε) 422 (5.34), 546 (4.18), 582 (3.70). MS (ESI): *m*/*z* 1022.3 M^+^˙.

#### 3.3.2. Synthesis of **2b**

The demetalation of **2a** (50 mg) was carried out with 10% H_2_SO_4_ in CH_2_Cl_2_. After about 5 min at room temperature, the reaction mixture was neutralized with an aqueous K_2_CO_3_ solution and extracted with dichloromethane. The organic layer was dried over Na_2_SO_4_ and, after evaporation of the solvent under reduced pressure, the residue was crystallized from dichloromethane/methanol. Porphyrin **2b** was obtained in quantitative yield.

^1^H NMR (300 MHz, CDCl_3_): δ −2.75 (s, 2H, *N*H), 5.95 (s, 1H, *N*H), 7.64–7.77 (m, 10H, *m*,*p*-Ph-H + 3-H), 7.82–7.84 (m, 3H, *m*,*p*-Ph-H), 8.14–8.23 (m, 8H, *o*-Ph-H), 8.62 (d, 1H, *J* = 4.8 Hz, β-H), 8.77–8.85 (m, 5H, β-H). ^19^F NMR (282 MHz, CDCl_3_): −159.9 to −160.3 (m, 1F), −159.3 (t, 2F, *J* = 21.2 Hz), −143.9 (d, 2F, *J* = 21.2 Hz). ^13^C NMR (75 MHz, CDCl_3_): 111.7, 116.8, 118.3, 120.2, 121.4, 126.1, 126.6, 126.7, 126.8, 126.9, 127.7, 127.8, 128.3, 128.6, 129.2, 132.8, 134.4, 134.5, 134.6, 134.7, 140.7, 142.0, 142.2, 142.5. δ. UV–Vis (CHCl_3_): λ_max_ (log ε) 419 (5.07), 522 (4.13), 559 (3.62), 594 (3.69), 650 (3.37). MS (ESI): *m*/*z* 796.5 [M + H]^+^. HRMS-ESI(+): *m*/*z* calcd. for C_50_H_31_N_5_F_5_ [M + H]^+^ 796.2494; found 796.2498.

#### 3.3.3. Synthesis of **4**

A solution of **2b** (20 mg, 25.2 µmol) in nitrobenzene (2 mL) was kept stirring under reflux for 72 h. The reaction mixture was poured on the top of a silica gel chromatography column, and the nitrobenzene was eluted with hexane. Then, the reaction product was eluted using a gradient of hexane/dichloromethane. The desired compound **4** was obtained in 53% yield (10.6 mg) after crystallization from dichloromethane and methanol.

^1^H NMR (500 MHz, CDCl_3_): δ −1.67 (s, 2H, *N*H), 7.50 (d, 1H, *J* = 7.9 Hz), 7.73–7.79 (m, 10H), 7.84 (s, 1H), 7.88 (t, 1H, *J* = 7.9 Hz), 8.15–8.19 (m, 4H), 8.26–8.28 (m, 2H), 8.66 (d, 1H, *J* = 4.6 Hz, β-H), 8.72 (d, 1H, *J* = 4.6 Hz, β-H), 8.74 (d, 1H, *J* = 4.9 Hz, β-H), 8.82 (d, 1H, *J* = 4.9 Hz, β-H), 8.90 (d, 1H, *J* = 4.7 Hz, β-H), 9.62 (d, 1H *J* = 7.9 Hz), 9.75 (d, 1H, *J* = 4.7 Hz, β-H). ^19^F NMR (282 MHz, CDCl_3_): δ −155.1 to −155.4 (m, 2F), −147.0 (t, *J* = 21.0 Hz, 1F), −139.1 to −139.4 (m, 2F). ^13^C NMR (125 MHz, CDCl_3_): δ 101.9, 109.7, 113.6, 117.7, 117.8, 119.1, 122.9, 123.2, 124.0, 124.5, 124.6, 126.77, 126.81, 127.0, 127.3, 127.5, 127.6, 127.7, 127.9, 128.5, 128.7, 133.1, 134.2, 134.5, 134.8, 134.9, 135.0, 135.8, 137.0, 138.4, 138.5, 142.1, 142.2, 142.3, 144.0, 146.7, 147.1, 147.7, 150.2, 152.2, 154.6, 156.0. UV–Vis (CHCl_3_): λ_max_ (log ε) 421 (5.26), 440 (5.19), 545 (4.15), 584 (4.41), 604 (4.21), 658 (4.18). MS (ESI): *m*/*z* 794.4 [M + H]^+^. HRMS-ESI(+): *m*/*z* calcd. for C_50_H_29_N_5_F_5_ [M + H]^+^ 794.2338; found 794.2349.

#### 3.3.4. General Procedure for **5a**,**b** and **6a**,**b**

Porphyrins **2b** or **4** (50 mg) were added to solutions of 3-sulfanylbenzoic acid or 4-sulfanylbenzoic acid (14.5 mg, 94 µmol, 1.5 eq.) in DMF (4 mL) and K_2_CO_3_ (36 mg, 260 µmol, 4 eq.). The mixtures were stirred at room temperature for 2 h or 8 h. Compounds **5a**,**b** and **6a**,**b** were obtained after precipitation from dichloromethane/methanol. **5a**: 36.8 mg, 63% yield; **5b**: 43.2 mg, 74% yield; **6a**: 43.3 mg, 74% yield; **6b**: 40.3 mg, 69% yield.

**5a**: ^1^H NMR (300 MHz, DMSO-*d_6_*, 40 °C): δ −2.81 (s, 2H, *N*H), 7.31 (d, 2H, *J* = 8.4 Hz), 7.62–7.70 (m, 3H), 7.78–7.85 (m, 10H), 7.92 (d, 2H, *J* = 8.4 Hz), 8.09–8.12 (m, 2H), 8.18–8.22 (m, 6H), 8.29 (s, 1H, *N*H), 8.62 (d, 1H, *J* = 4.9 Hz, β-H), 8.73–8.85 (m, 5H, β-H). ^19^F NMR (282 MHz, DMSO-*d_6_*): −132.1 (d, 2F, *J* = 19.0 Hz), −149.0 (d, 2F, *J* = 19.0 Hz). ^13^C NMR (125 MHz, DMSO-*d_6_*): δ 118.9, 119.5, 119.8, 120.8, 121.3, 126.6, 127.5, 127.6, 127.7, 128.6, 130.8, 133.7, 133.9, 134.5, 134.65, 134.72, 139.9, 141.3, 141.7, 141.9, 162.8, 167.5. UV–Vis (CHCl_3_): λ_max_ (log ε) 424 (5.26), 523 (4.31), 554 (3.89), 594 (3.86), 651 (3.48). MS (ESI): *m*/*z* 930.4 [M + H]^+^. HRMS-ESI(+): *m*/*z* calcd. for C_57_H_36_F_4_N_5_O_2_S [M + H]^+^ 930.2556; found 930.2559.

**5b**: ^1^H NMR (300 MHz, DMSO-*d_6_*, 40 °C): δ −2.82 (s, 2H, *N*H), 6.58 (s, 1H, *N*H), 7.40–7.45 (m, 2H), 7.56–7.66 (m, 4H), 7.78–7.86 (m, 11H), 8.06 (d, 2H, *J* = 6.7 Hz), 8.17–8.22 (m, 6H), 8.29 (s, 1H), 8.59 (d, 1H, *J* = 4.9 Hz, β-H), 8.73–8.85 (m, 5H, β-H). ^19^F NMR (282 MHz, DMSO-*d_6_*): −132.4 (d, 2F, *J* = 19.4 Hz), −149.4 (d, 2F, *J* = 19.4 Hz). ^13^C NMR (125 MHz, DMSO-*d_6_*): δ 118.9, 119.5, 120.5, 121.2, 127.5, 127.6, 128.3, 128.6, 128.9, 129.7, 130.8, 133.6, 134.5, 134.6, 134.7, 139.8, 141.3, 141.7, 141.9, 142.0, 143.2, 146.1, 146.7, 146.8, 162.4, 168.4, 186.6. UV–Vis (CHCl_3_): λ_max_ (log ε) 415 (5.03), 424 (5.03), 522 (4.13), 550 (3.79), 594 (3.72), 650 (3.35). MS (ESI): *m*/*z* 930.4 [M + H]^+^. HRMS-ESI(+): *m*/*z* calcd. for C_57_H_36_O_2_N_5_F_4_S [M + H]^+^ 930.2520; found 930.2530.

**6a**: ^1^H NMR (300 MHz, CDCl_3_): δ −1.63 (s, 2H, *N*H), 7.50 (d, 2H, *J* = 8.3 Hz), 7.73–7.75 (m, 4H), 7.78–7.85 (m, 8H), 8.15–8.20 (m, 8H), 8.25–8.29 (m, 2H), 8.66 (d, 1H, *J* = 4.6 Hz, β-H), 8.72 (d, 1H, *J* = 4.6 Hz, β-H), 8.78 (d, 1H, *J* = 5.0 Hz, β-H), 8.83 (d, 1H, *J* = 5.0 Hz, β-H), 8.90 (d, 1H, *J* = 4.8 Hz, β-H), 9.64 (dd, 1H, *J* = 7.9 and 1.0 Hz), 9.77 (d, 1H, *J* = 4.8 Hz, β-H). ^19^F NMR (282 MHz, CDCl_3_): δ −137.8 (dd, *J* = 21.6 and 9.0 Hz, 2F), −125.5 (dd, *J* = 21.6 and 9.0 Hz, 2F). ^13^C NMR (125 MHz, CDCl_3_): δ 102.1, 109.7, 113.8, 117.7, 117.8, 118.4, 122.8, 123.16, 123.24, 124.6, 126.77, 126.81, 127.0, 127.3, 127.6, 127.8, 127.9, 128.5, 128.66, 128.72, 129.1, 131.2, 131.3, 132.7, 133.2, 134.3, 134.5, 134.8, 134.9, 135.8, 138.8, 139.7, 142.0, 142.2, 142.3, 143.6, 162.1, 168.6. UV–Vis (CHCl_3_): λ_max_ (log ε) 425 (4.46), 440 (4.48), 544 (3.52), 584 (3.74), 605 (3.59), 658 (3.59). MS (ESI): *m*/*z* 928.4 [M + H]^+^. HRMS-ESI(+): *m*/*z* calcd. for C_57_H_34_F_4_N_5_O_2_S [M + H]^+^ 928.2364; found 928.2374.

**6b**: ^1^H NMR (300 MHz, CDCl_3_): δ −1.67 (s, 2H, *N*H), 7.52–7.56 (m, 2H), 7.72–7.78 (m, 9H), 7.85–7.87 (m, 2H), 7.92–7.96 (m, 2H), 8.14–8.19 (m, 6H), 8.24–8.27 (m, 3H), 8.33 (s, 1H), 8.65 (d, 1H, *J* = 4.6 Hz, β-H), 8.71 (d, 1H, *J* = 4.6 Hz, β-H), 8.74 (d, 1H, *J* = 5.1 Hz, β-H), 8.82 (d, 1H, *J* = 5.1 Hz, β-H), 8.88 (d, 1H, *J* = 4.7 Hz, β-H), 9.60 (d, 1H, *J* = 8.3 Hz), 9.74 (d, 1H, *J* = 4.7 Hz, β-H). ^19^F NMR (282 MHz, CDCl_3_): δ −138.6 (dd, *J* = 18.4 and 8.6 Hz, 2F), −126.4 (dd, *J* = 18.4 and 8.6 Hz, 2F). ^13^C NMR (125 MHz, CDCl_3_): δ 113.7, 113.9, 118.3, 123.2, 126.76, 126.80, 127.0, 127.3, 127.5, 127.6, 127.7, 127.8, 128.2, 128.48, 128.51, 128.6, 128.7, 129.1, 130.1, 132.7, 134.3, 134.8, 135.8, 136.2, 142.1. UV–Vis (CHCl_3_): λ_max_ (log ε) 424 (4.43), 444 (4.50), 544 (3.46), 585 (3.71), 605 (3.54), 658 (3.52). MS (ESI): *m*/*z* 928.4 [M + H]^+^. HRMS-ESI(+): *m*/*z* calcd. for C_57_H_34_F_4_N_5_O_2_S [M + H]^+^ 928.2364; found 928.2381.

#### 3.3.5. Synthesis of **5c**

A solution of **5b** (10 mg, 10.8 µmol) and Zn(OAc)_2_ (19.7 mg, 108 µmol, 10 equiv.) in a mixture of chloroform (3 mL) and methanol (1 mL) was heated at reflux for 15 min. After cooling, the solvents were removed under reduced pressure. The solid residue was dissolved in chloroform and the organic layer was washed with water and dried over Na_2_SO_4_. The Zn(II) porphyrin **5c** was obtained in 98% yield (10.5 mg) after crystallization from dichloromethane and methanol.

^19^F NMR (282 MHz, CDCl_3_): δ −150.0 (d, *J* = 20.9 Hz, 2F), −132.8 (d, *J* = 20.9 Hz, 2F). UV–Vis (CHCl_3_): λ_max_ (log ε) 430 (4.74), 558 (3.73), 592 (3.22), 629 (2.87). MS (ESI): *m*/*z* 991.3 M^+^˙. HRMS-ESI(+): *m*/*z* calcd. for C_57_H_34_O_2_N_5_F_4_SZn [M + H]^+^ 992.1656; found 992.1655.

### 3.4. Differential Pulse Voltammetry (DPV)

Differential pulse voltammetry (DPV) voltammograms were measured on a μAutolab Type III potentiostat/galvanostat (Metrohm Autolab B. V., Utrecht, The Netherlands), supervised by the GPES (General Purpose Electrochemical System) program version 4.9 (Eco-Chemie, B. V. Software, Utrecht, The Netherlands). The electrolytic cell, in which three electrodes can be placed together, had a volume capacity of 5 mL. A saturated calomel reference electrode (SCE, saturated KCl; Metrohm, Utrecht, The Netherlands) was used as the standard electrode. A glassy carbon electrode (f = 1.0 mm, BAS Inc., West Lafayette, IN, USA) was the chosen working electrode. The counter-electrode consisted of Pt wire. The working electrode was polished before use on 2–7/″ micro-cloth (Buehler) polishing pads using 1.0 and 0.3 mm alumina–water slurry (Buehler, Esslingen, Germany), then cleaned with water and ethanol. This desorption method was always repeated before carrying out electrochemical measurements. The electrolyte solution contained the porphyrin dye (0.5 mM) and the supporting electrolyte tetrabutylammonium hexafluorophosphate (0.1 M, TBAPF_6_) dissolved in dry dichloromethane. The electrolyte solutions were degassed by purging N_2_ before each measurement. The voltammograms were recorded at three different scan rates (10, 20, and 30 mV s^−1^). The points of intersection between the tangent lines of the rising current and the baseline current were regarded as the onset values.

### 3.5. DSSC Fabrication and Photovoltaic Characterization

The conductive FTO-glass (TEC7, Greatcell Solar, Queanbeyan, Australia) employed for preparing transparent electrodes underwent meticulous cleaning with detergent followed by thorough rinsing with water and ethanol. For the anode preparation, the conductive glass plates (measuring 15 cm × 4 cm) were immersed in a TiCl_4_/water solution (40 mM) at 70 °C for 30 min, then washed with water and ethanol before undergoing sintering at 500 °C for 30 min. This precise sequence is crucial for enhancing adherence of subsequent nanocrystalline layers and establishing a ‘blocking-layer’ to decrease charge recombination between electrons in the FTO and holes in the I^−^/I_3_^−^ redox couple.

Subsequently, the TiO_2_ nanocrystalline layers were deposited onto these pre-treated FTO plates through screen-printing with transparent titania paste (18NR-T, Greatcell Solar) using a polyester fiber frame with 43.80 mesh per cm^2^. This dual-step process, involving coating and drying at 125 °C, was iterated twice. The TiO_2_-coated plates underwent gradual heating up to 325 °C, followed by a temperature increase to 375 °C within 5 min, then further to 500 °C for sintering over 30 min, concluding with cooling to room temperature. A second treatment with the same TiCl_4_/water solution (40 mM) was executed, following the aforementioned procedure, serving as an optimization step to augment surface roughness for improved dye adsorption, thereby positively influencing photocurrent generation under illumination.

Finally, a layer of reflective titania paste (WER2-O, Greatcell Solar) was screen-printed and sintered at 500 °C. This layer comprising anatase particles sized 150–200 nm functions as a ‘photon-trapping’ layer, further enhancing photocurrent. Each anode was precisely cut into rectangular pieces (measuring 2 cm × 1.5 cm) with a spot area of 0.196 cm^2^ and a thickness of 15 μm. These prepared anodes were then immersed for 16 h in a 0.5 mM dye solution in dichloromethane at room temperature in darkness, followed by removal of excess dye via rinsing with the same solvent.

For investigating the impact of chenodeoxycholic acid (CDCA) addition on the photovoltaic properties, the adsorption process was repeated for each compound with a 0.5 mM dye solution prepared in dichloromethane with 50 mM CDCA.

Each counter-electrode comprised an FTO-glass plate (measuring 2 cm × 2 cm) with a 1.0 mm diameter hole drilled. These perforated substrates were meticulously cleaned with water and ethanol to eliminate residual glass powder and organic contaminants. Transparent Pt catalyst (PT1, Greatcell Solar) was deposited on the conductive face of the FTO-glass using a doctor blade technique. A strip of adhesive tape (3M Magic, Springfield, IL, USA) was applied to one edge of the glass plate to control film thickness and mask an electric contact strip. After uniform spreading of Pt paste on the substrate using a glass rod along the tape spacer, the adhesive tape strip was removed, and the glasses were heated at 550 °C for 30 min.

The photoanode and Pt counter-electrode were then assembled into a sandwich-type arrangement and sealed using a hot melt gasket made of Surlyn ionomer (Meltonix 1170-25, Solaronix SA, Aubonne, Switzerland) via a thermopress. The electrolyte, comprising the redox couple, I^−^/I_3_^−^ (0.8 M LiI and 0.05 M I_2_), dissolved in an acetonitrile/pentanenitrile (85:15, % *v*/*v*) mixture, was introduced into the cell via backfilling under vacuum through the hole drilled in the back of the cathode, which was subsequently sealed with adhesive tape. For each compound, a minimum of two cells were assembled under identical conditions, and the efficiencies were measured five times for each cell, resulting in a minimum of ten measurements per compound.

Current–Voltage curves were recorded using a digital Keithley SourceMeter multimeter (PVIV-1A) (Newport, M. T. Brandão, Porto, Portugal) connected to a PC. Simulated sunlight irradiation was provided by an Oriel solar simulator (Model LCS-100 Small Area Sol1A, 300 W Xe Arc lamp equipped with AM 1.5 filter, 100 mW/cm^2^) (Newport, M. T. Brandão). The thickness of the oxide film deposited on the photoanodes was measured using an Alpha-Step D600 Stylus Profiler (KLA-Tencor, Milpitas, CA, USA).

## 4. Conclusions

In this study, 2-amino-substituted porphyrin derivatives suitably functionalized with a carboxy group were prepared and evaluated as dyes in DSSC. The new compounds were successfully synthesized in two or three steps, in good overall yields, by nucleophilic aromatic substitution reactions with 3-sulfanyl- and 4-sulfanylbenzoic acid on porphyrins bearing a pentafluorophenyl group. Electrochemical studies revealed that the HOMO and LUMO energy levels are consistent with the requirements for effective electron transfer and dye regeneration, indicating that these dyes fit as suitable candidates for DSSC devices. However, the DSSC devices fabricated with these compounds revealed performances between 0.4% and 10% of the value obtained for N719. This low performance can be primarily attributed to their reduced open-circuit voltage and short-circuit current density values. One of the reasons for the reduced PCE values seems to arise from porphyrin aggregation phenomena, as demonstrated by the increased efficiencies in the presence of the disaggregating agent CDCA.

## Figures and Tables

**Table 1:** Absorption and emission data of compounds **5a**–**c** and **6a**,**b** in chloroform.

Compounds	λ_max,abs_ (nm) ^a^	log ε (M^−1^ cm^−1^)	λ_max,em_ (nm) ^b^	Stokes Shift (cm^−1^) ^c^
**5a**	424	5.26	655 719	94
	523	4.31		
	554	3.89		
	594	3.86		
	651	3.48		
**5b**	415	5.03	657	164
	424	5.03719		
	522	4.13		
	550	3.79		
	594	3.72		
	650	3.35		
**5c**	430	4.74	608	—
	558	3.73		
	592 sh	3.22	658	
	629 sh	2.87		
**6a**	425	4.46	661	69
	440	4.48		
	544	3.52		
	584	3.74	727	
	605	3.59		
	658	3.59		
**6b**	424	4.43	661	69
	444	4.50		
	544	3.46		
	585	3.71	728	
	605	3.54		
	658	3.52		

^a^ 10^−5^–10^−6^ mol L^−1^ in CHCl_3_. ^b^ Excitation at maximum absorption. ^c^ Q4 band.

**Table 2:** Electrochemical properties of porphyrin derivatives **5** and **6** obtained from differential pulse voltammetry measurements in CH_2_Cl_2_ solution with a dye concentration of 0.5 mM and 0.1 M TBAPF_6_ at scan rates of 10, 20, and 30 mV s^−1^.

Dye	E_ox_ SCE (V)	HOMO vs. Vacuum (eV)	E_red_ SCE (V)	LUMO vs. Vacuum (eV)	Band Gap (eV)
**5a**	0.819	−5.26	−1.063	−3.38	1.88
**5b**	0.761	−5.20	−1.151	−3.29	1.91
**5c**	0.649	−5.09	−1.210	−3.23	1.86
**6a**	0.638	−5.08	−1.068	−3.37	1.71
**6b**	0.677	−5.12	−0.995	−3.45	1.67

**Table 3:** Photovoltaic performance of the porphyrin derivatives 5 and 6 and N719 under 100 mW cm^−2^ simulated AM 1.5 G illumination, using 0.8 M LiI and 0.05 M I_2_ in acetonitrile/pentanenitrile (85:15, % *v*/*v*) as electrolyte.

Compound	V_oc_ (mV)	J_sc_ (mA cm^−2^)	FF	PCE (%)
**5a**	213 ± 4	0.30 ± 0.02	0.41 ± 0.08	0.03 ± 0.01
**5b**	206 ± 25	0.23 ± 0.03	0.43 ± 0.12	0.02 ± 0.01
**5c**	361 ± 7	1.8 ± 0.1	0.56 ± 0.06	0.37 ± 0.06
**6a**	185 ± 5	0.17 ± 0.03	0.42 ± 0.03	0.013 ± 0.003
**6b**	280 ± 8	0.35 ± 0.01	0.49 ± 0.05	0.049 ± 0.003
**N719**	427 ± 12	15.3 ± 0.5	0.57 ± 0.01	3.70 ± 0.09

**Table 4:** Comparison of the photovoltaic performance under 100 mW cm^−2^ simulated AM 1.5G illumination for DSSC based on compound **5a** adsorbed from 0.5 mM solutions in dichloromethane, and **5a** plus 50 mM CDCA, using 0.8 M LiI and 0.05 M I_2_ in acetonitrile/pentanenitrile (85:15, % *v*/*v*) as electrolyte.

Compound	V_oc_ (mV)	J_sc_ (mA cm^−2^)	FF	PCE (%)
**5a**	213 ± 4	0.30 ± 0.02	0.41 ± 0.08	0.03 ± 0.01
**5a** **+ CDCA**	329 ± 7	0.53 ± 0.02	0.63 ± 0.01	0.11 ± 0.01

## Data Availability

New data are available in the Supplementary Materials.

## Supplementary Materials

The supporting information can be downloaded at: https://www.mdpi.com/article/10.3390/ijms25115979/s1.

## References

[1] Higashino T., Imahori H. (2015). Porphyrins as Excellent Dyes for Dye-Sensitized Solar Cells: Recent Developments and Insights. Dalt. Trans..

[2] Ji J.-M., Zhou H., Kim H.K. (2018). Rational Design Criteria for D–π–A Structured Organic and Porphyrin Sensitizers for Highly Efficient Dye-Sensitized Solar Cells. J. Mater. Chem. A.

[3] Jin Z., Masuda H., Yamanaka N., Minami M., Nakamura T., Nishikitani Y. (2009). Efficient Electron Transfer Ruthenium Sensitizers for Dye-Sensitized Solar Cells. J. Phys. Chem. C.

[4] Jella T., Srikanth M., Bolligarla R., Soujanya Y., Singh S.P., Giribabu L. (2015). Benzimidazole-Functionalized Ancillary Ligands for Heteroleptic Ru(II) Complexes: Synthesis, Characterization and Dye-Sensitized Solar Cell Applications. Dalt. Trans..

[5] Jia H.-L., Li S.-S., Peng Z.-J., Zhao J., Gong B.-Q., Guan M.-Y., Zheng H.-G., Xia F.-F. (2020). Efficient Phenothiazine-Ruthenium Sensitizers with High Open-Circuit Voltage (Voc) for High Performance Dye-Sensitized Solar Cells. Dye. Pigment..

[6] Wu Y., Zhu W. (2013). Organic Sensitizers from D–π–A to D–A–π–A: Effect of the Internal Electron-Withdrawing Units on Molecular Absorption, Energy Levels and Photovoltaic Performances. Chem. Soc. Rev..

[7] Zhang L., Yang X., Wang W., Gurzadyan G.G., Li J., Li X., An J., Yu Z., Wang H., Cai B. (2019). 13.6% Efficient Organic Dye-Sensitized Solar Cells by Minimizing Energy Losses of the Excited State. ACS Energy Lett..

[8] Shen Z., Zhang X., Giordano F., Hu Y., Hua J., Zakeeruddin S.M., Tian H., Grätzel M. (2017). Significance of π-Bridge Contribution in Pyrido[3,4-*b*]Pyrazine Featured D-A-π-A Organic Dyes for Dye-Sensitized Solar Cells. Mater. Chem. Front..

[9] Jiao Y., Liu S., Shen Z., Mao L., Ding Y., Ren D., Eickemeyer F.T., Pfeifer L., Cao D., Xu W. (2021). Carbazol-Phenyl-Phenothiazine-Based Sensitizers for Dye-Sensitized Solar Cells. J. Mater. Chem. A.

[10] Zeng K., Tong Z., Ma L., Zhu W.H., Wu W., Xie Y., Xie Y. (2020). Molecular Engineering Strategies for Fabricating Efficient Porphyrin-Based Dye-Sensitized Solar Cells. Energy Environ. Sci..

[11] Sekaran B., Misra R. (2022). β-Pyrrole Functionalized Porphyrins: Synthesis, Electronic Properties, and Applications in Sensing and DSSC. Coord. Chem. Rev..

[12] Ramasamy S., Bhagavathiachari M., Suthanthiraraj S.A., Pichai M. (2022). Mini Review on the Molecular Engineering of Photosensitizer: Current Status and Prospects of Metal-Free/Porphyrin Frameworks at the Interface of Dye-Sensitized Solar Cells. Dye. Pigment..

[13] Hu Y., Alsaleh A., Trinh O., D’Souza F., Wang H. (2021). β-Functionalized Push–Pull opp-Dibenzoporphyrins as Sensitizers for Dye-Sensitized Solar Cells: The Push Group Effect. J. Mater. Chem. A.

[14] Chen C., Chen J., Nguyen V.S., Wei T., Yeh C. (2021). Double Fence Porphyrins That Are Compatible with Cobalt(II/III) Electrolyte for High-Efficiency Dye-Sensitized Solar Cells. Angew. Chemie Int. Ed..

[15] He J., Wang B., Chang S., Chen T. (2015). Ruthenium-Based Photosensitizers for Dye-Sensitized Solar Cells. Organometallics and Related Molecules for Energy Conversion.

[16] Prima E.C., Nugroho H.S., Nugraha, Refantero G., Panatarani C., Yuliarto B. (2020). Performance of the Dye-Sensitized Quasi-Solid State Solar Cell with Combined Anthocyanin-Ruthenium Photosensitizer. RSC Adv..

[17] Grätzel M. (2003). Dye-Sensitized Solar Cells. J. Photochem. Photobiol. C Photochem. Rev..

[18] Hagfeldt A., Boschloo G., Sun L., Kloo L., Pettersson H. (2010). Dye-Sensitized Solar Cells. Chem. Rev..

[19] O’Regan B., Grätzel M. (1991). A Low-Cost, High-Efficiency Solar Cell Based on Dye-Sensitized Colloidal TiO2 Films. Nature.

[20] Song H., Liu Q., Xie Y. (2018). Porphyrin-Sensitized Solar Cells: Systematic Molecular Optimization, Coadsorption and Cosensitization. Chem. Commun..

[21] Di Carlo G., Biroli A.O., Tessore F., Caramori S., Pizzotti M. (2018). β-Substituted Zn(II) Porphyrins as Dyes for DSSC: A Possible Approach to Photovoltaic Windows. Coord. Chem. Rev..

[22] Bhuse D.V., Bhuse V.M., Bhagat P.R. (2022). Ant-like Small Molecule Metal-Free Dimeric Porphyrin Sensitizer for True Energy-Generating DSSC with 9.3% Efficiency. J. Mater. Sci. Mater. Electron..

[23] Mathew S., Yella A., Gao P., Humphry-Baker R., Curchod B.F.E., Ashari-Astani N., Tavernelli I., Rothlisberger U., Nazeeruddin M.K., Grätzel M. (2014). Dye-Sensitized Solar Cells with 13% Efficiency Achieved through the Molecular Engineering of Porphyrin Sensitizers. Nat. Chem..

[24] Carella A., Borbone F., Centore R. (2018). Research Progress on Photosensitizers for DSSC. Front. Chem..

[25] Li L.L., Diau E.W.G. (2013). Porphyrin-Sensitized Solar Cells. Chem. Soc. Rev..

[26] Hombrecher H.K., Gherdan V.M., Ohm S., Cavaleiro J.A.S., da Graça M., Neves P.M.S., de Fátima Condesso M. (1993). Synthesis and Electrochemical Investigation of β-Alkyloxy Substituted meso-Tetraphenylporphyrins. Tetrahedron.

[27] Costa J.I.T., Farinha A.S.F., Neves M.G.P.M.S., Tomé A.C. (2016). An Easy Access to Porphyrin Triads and Their Supramolecular Interaction with a Pyridyl [60]Fulleropyrrolidine. Dye. Pigment..

[28] Pereira A.M.V.M., Alonso C.M.A., Neves M.G.P.M.S., Tomé A.C., Silva A.M.S., Paz F.A.A., Cavaleiro J.A.S. (2008). A New Synthetic Approach to *N*-Arylquinolino[2,3,4-*at*]Porphyrins from β-Arylaminoporphyrins. J. Org. Chem..

[29] Castro K.A.D.F., Moura N.M.M., Figueira F., Ferreira R.I., Simões M.M.Q., Cavaleiro J.A.S., Faustino M.A.F., Silvestre A.J.D., Freire C.S.R., Tomé J.P.C. (2019). New Materials Based on Cationic Porphyrins Conjugated to Chitosan or Titanium Dioxide: Synthesis, Characterization and Antimicrobial Efficacy. Int. J. Mol. Sci..

[30] Diskin-Posner Y., Patra G.K., Goldberg I. (2001). Supramolecular Assembly of Metalloporphyrins in Crystals by Axial Coordination through Amine Ligands. J. Chem. Soc. Dalt. Trans..

[31] Bravin C., Badetti E., Licini G., Zonta C. (2021). Tris(2-Pyridylmethyl)Amines as Emerging Scaffold in Supramolecular Chemistry. Coord. Chem. Rev..

[32] Arnaut L.G. (2011). Design of Porphyrin-Based Photosensitizers for Photodynamic Therapy. Adv. Inorg. Chem..

[33] Cardona C.M., Li W., Kaifer A.E., Stockdale D., Bazan G.C. (2011). Electrochemical Considerations for Determining Absolute Frontier Orbital Energy Levels of Conjugated Polymers for Solar Cell Applications. Adv. Mater..

[34] Fernandes S.S.M., Castro M.C.R., Pereira A.I., Mendes A., Serpa C., Pina J., Justino L.L.G., Burrows H.D., Raposo M.M.M. (2017). Optical and Photovoltaic Properties of Thieno[3,2-b]thiophene-Based Push-Pull Organic Dyes with Different Anchoring Groups for Dye-Sensitized Solar Cells. ACS Omega.

[35] Hart A.S., KC C.B., Gobeze H.B., Sequeira L.R., D’Souza F. (2013). Porphyrin-Sensitized Solar Cells: Effect of Carboxyl Anchor Group Orientation on the Cell Performance. ACS Appl. Mater. Interfaces.

[36] Nasrollahi R., Martín-Gomis L., Fernández-Lázaro F., Zakavi S., Sastre-Santos Á. (2019). Effect of the Number of Anchoring and Electron-Donating Groups on the Efficiency of Free-Base- and Zn-Porphyrin-Sensitized Solar Cells. Materials.

[37] Zou J., Tang Y., Baryshnikov G., Yang Z., Mao R., Feng W., Guan J., Li C., Xie Y. (2022). Porphyrins Containing a Tetraphenylethylene-Substituted Phenothiazine Donor for Fabricating Efficient Dye Sensitized Solar Cells with High Photovoltages. J. Mater. Chem. A.

[38] Kurumisawa Y., Higashino T., Nimura S., Tsuji Y., Iiyama H., Imahori H. (2019). Renaissance of Fused Porphyrins: Substituted Methylene-Bridged Thiophene-Fused Strategy for High-Performance Dye-Sensitized Solar Cells. J. Am. Chem. Soc..

[39] Xie M., Liu J., Dai L., Peng H., Xie Y. (2023). Advances and Prospects of Porphyrin Derivatives in the Energy Field. RSC Adv..

[40] Zhang Y., Higashino T., Imahori H. (2023). Molecular Designs, Synthetic Strategies, and Properties for Porphyrins as Sensitizers in Dye-Sensitized Solar Cells. J. Mater. Chem. A.

[41] Armarego W.L.F., Chai C. (2013). Purification of Laboratory Chemicals.

