# Supplementary material for: Synthesis and Photovoltaic Performance of β-Amino-Substituted Porphyrin Derivatives
Source: Int J Mol Sci. 2024 May 29;25(11):5979. doi: 10.3390/ijms25115979 (PMC11172761; doi:10.3390/ijms25115979)
Supplement: Supplementary file 1 [file ijms-25-05979-s001.zip › ijms-2961537-supplementary/ijms-2961537-supplementary.pdf]

Synthesis and Photovoltaic Performance of β-Amino-substituted Porphyrin Derivatives

Ana F. R. Cerqueira[1], Ana Lucia Pinto[2], Gabriela Malta[2], Maria G.P.M.S. Neves[1], A. Jorge Parola[2,*], Augusto C. Tomé[1,*]

[1] LAQV-REQUIMTE, Department of Chemistry, University of Aveiro, 3810-193 Aveiro, Portugal

[2] LAQV-REQUIMTE, Department of Chemistry, NOVA School of Science and Technology, FCT NOVA, Universidade NOVA de Lisboa, 2829-516 Caparica, Portugal

INDEX

NMR, Mass and UV-Vis spectra of compounds 1

Differential Pulse Voltammetry Results 29

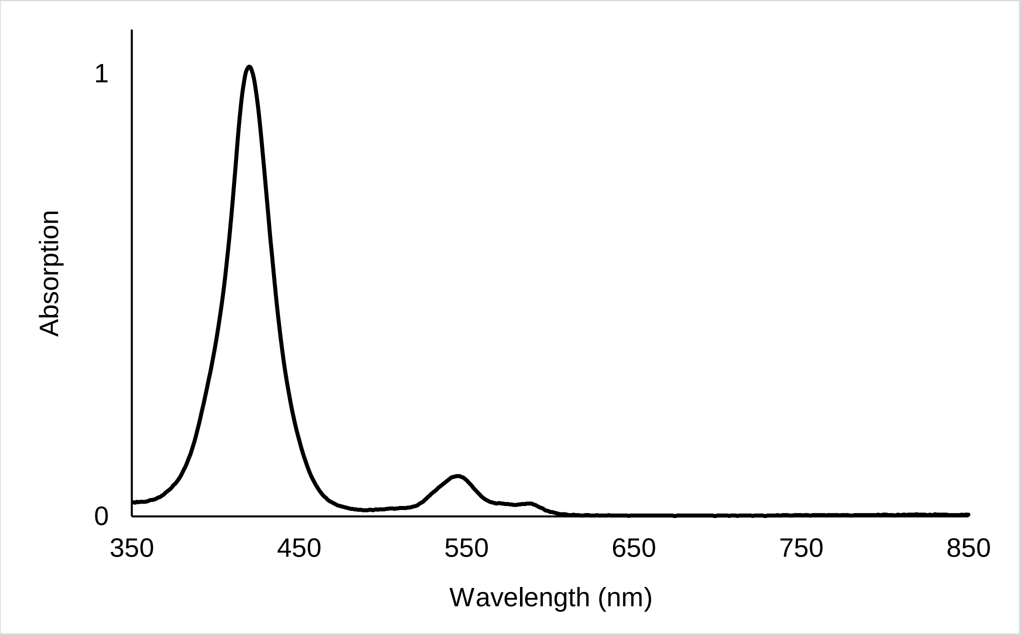

Figure S1 Absorption spectrum of compound **2a** in $CHCl_3$ ($2.4x10^{-5}$ mol.L^{-1}).

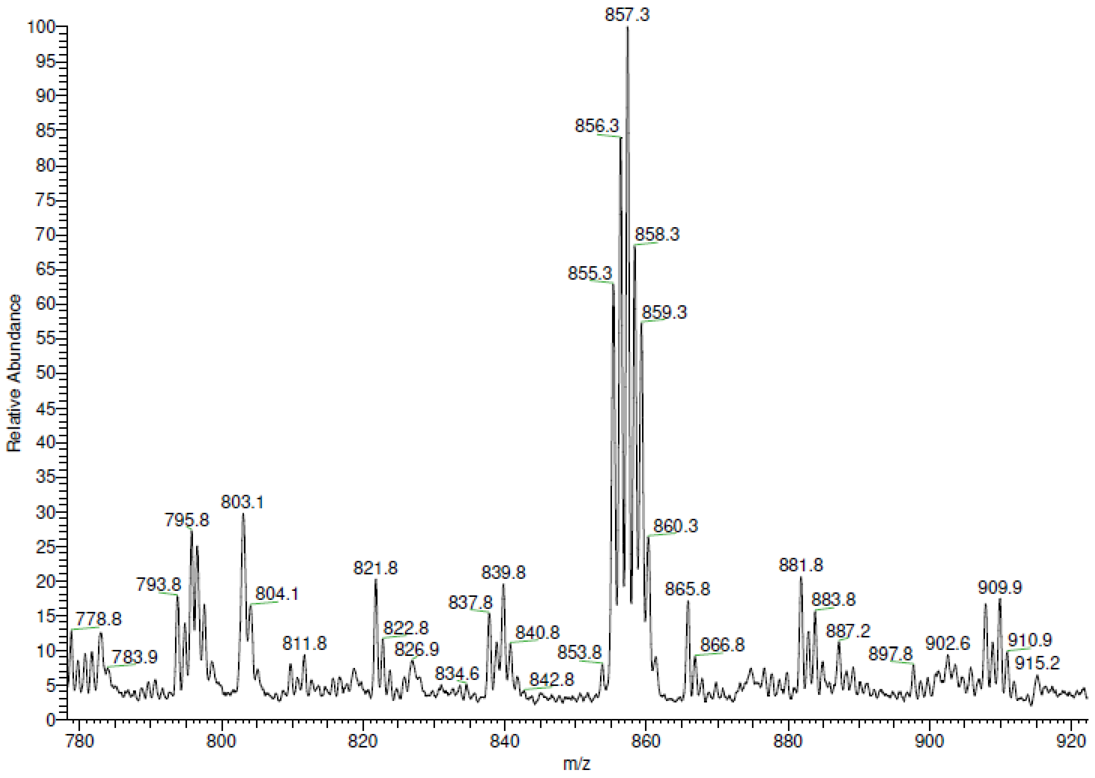

Figure S2. MS-ESI(+) spectrum of **2a**.

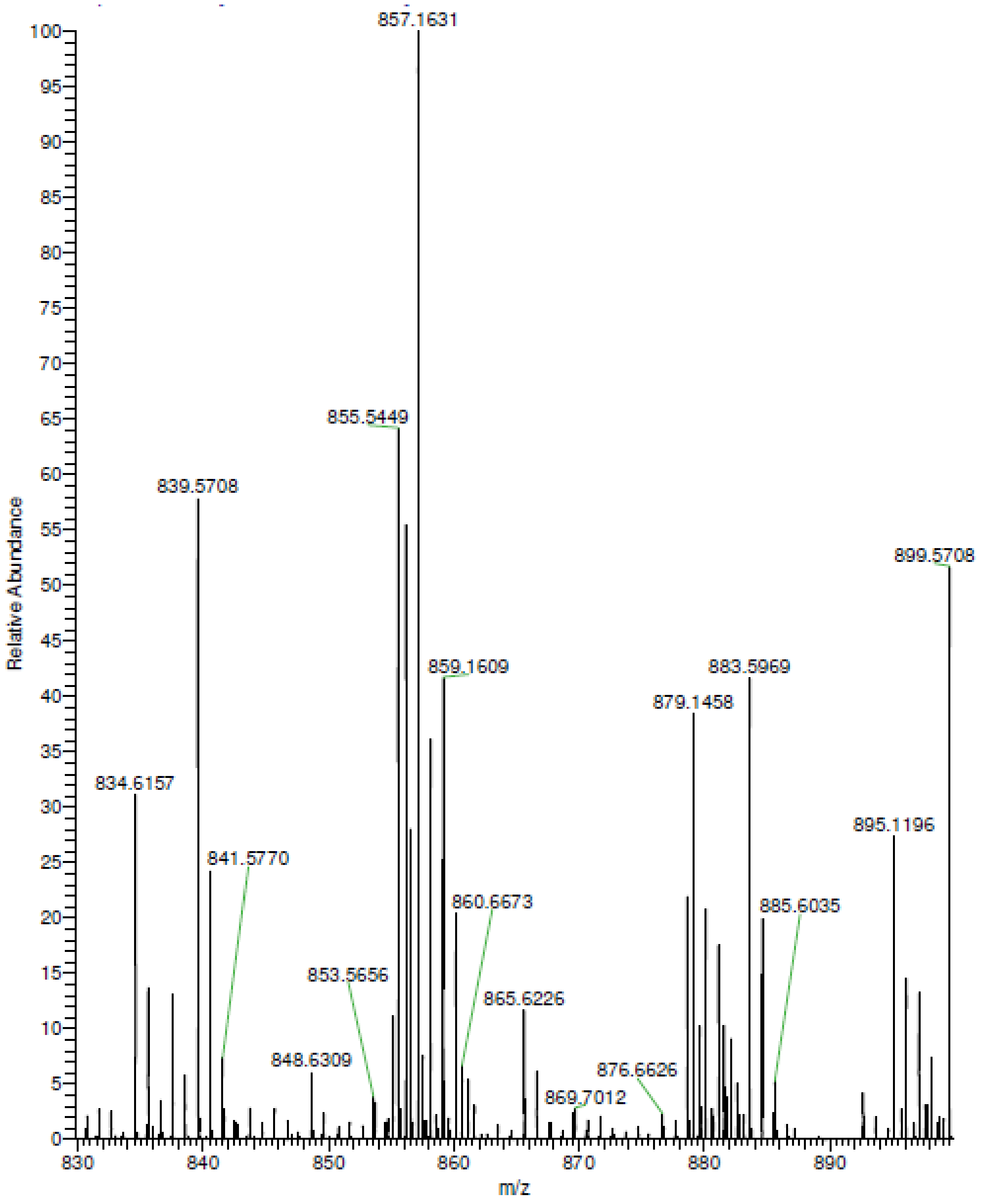

Figure S3. HRMS-ESI(+) spectrum of **2a**.

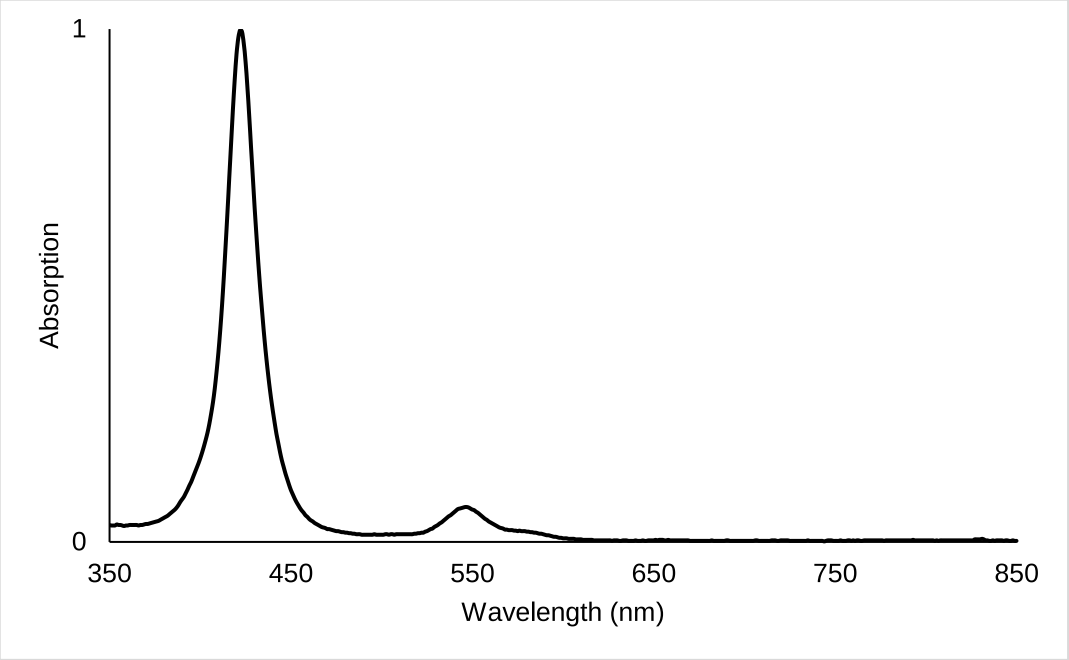

Figure S4 Absorption spectrum of compound **3** in $CHCl_3$ ($4.5x10^{-6}$ mol.L^{-1}).

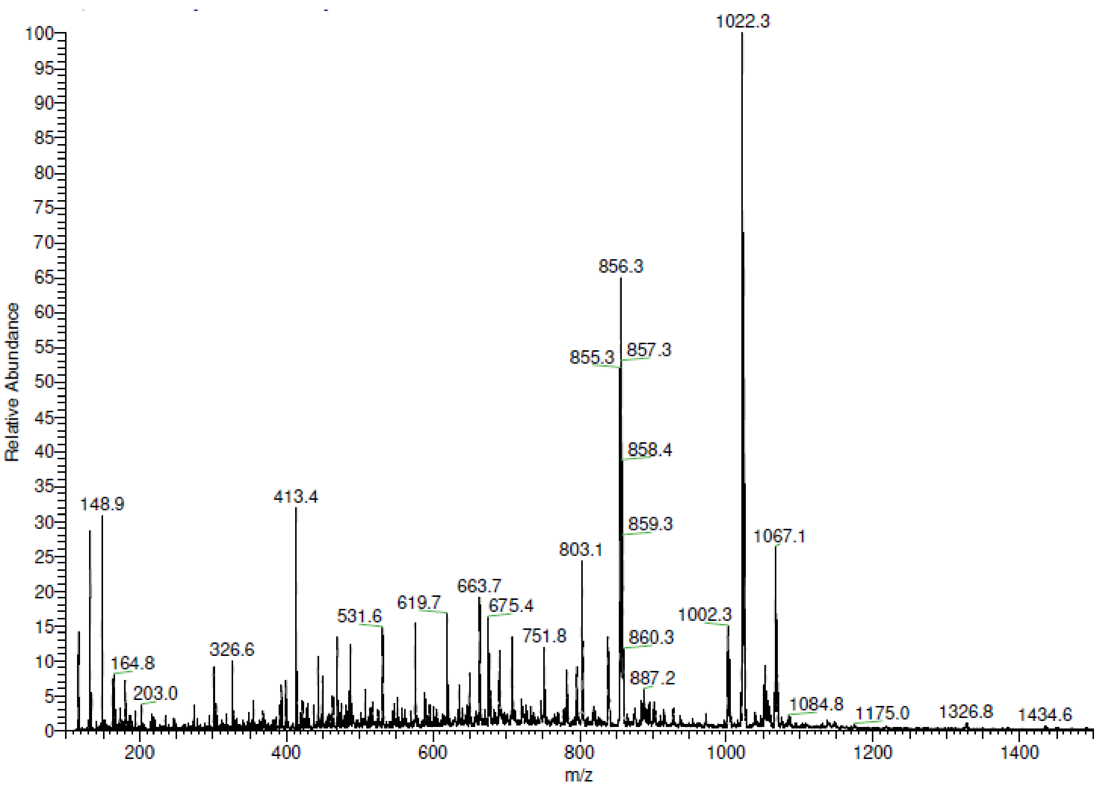

Figure S5. MS-ESI(+) spectrum of **3**.

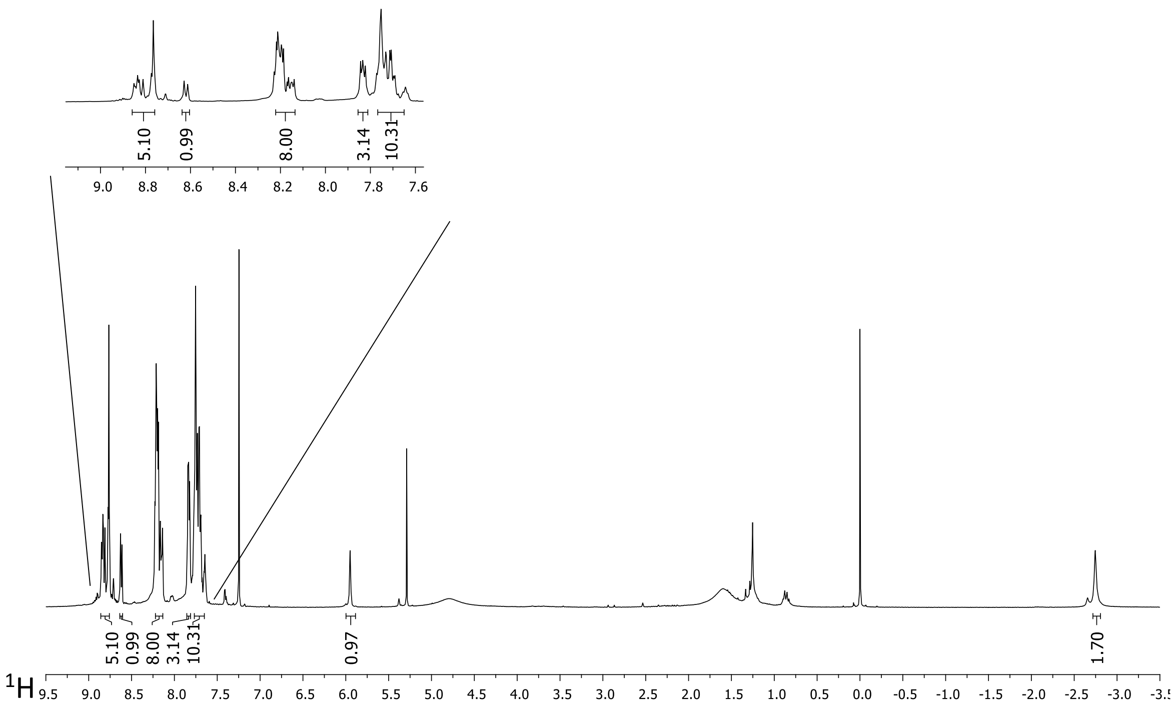

Figure S6. ^{1}H NMR spectrum of **2b** in $CDCl_3$ (300

MHz).

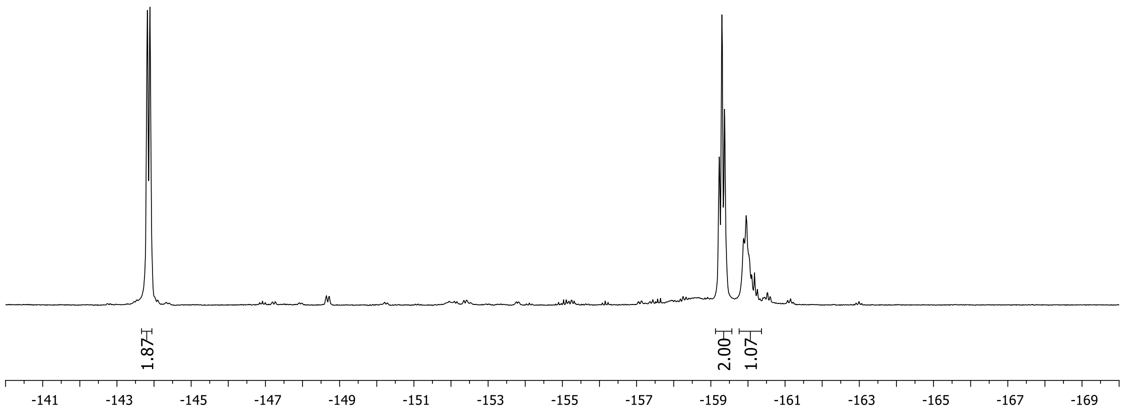

Figure S7. ^{19}F NMR spectrum of compound **2b** in $CDCl_3$ (282 MHz).

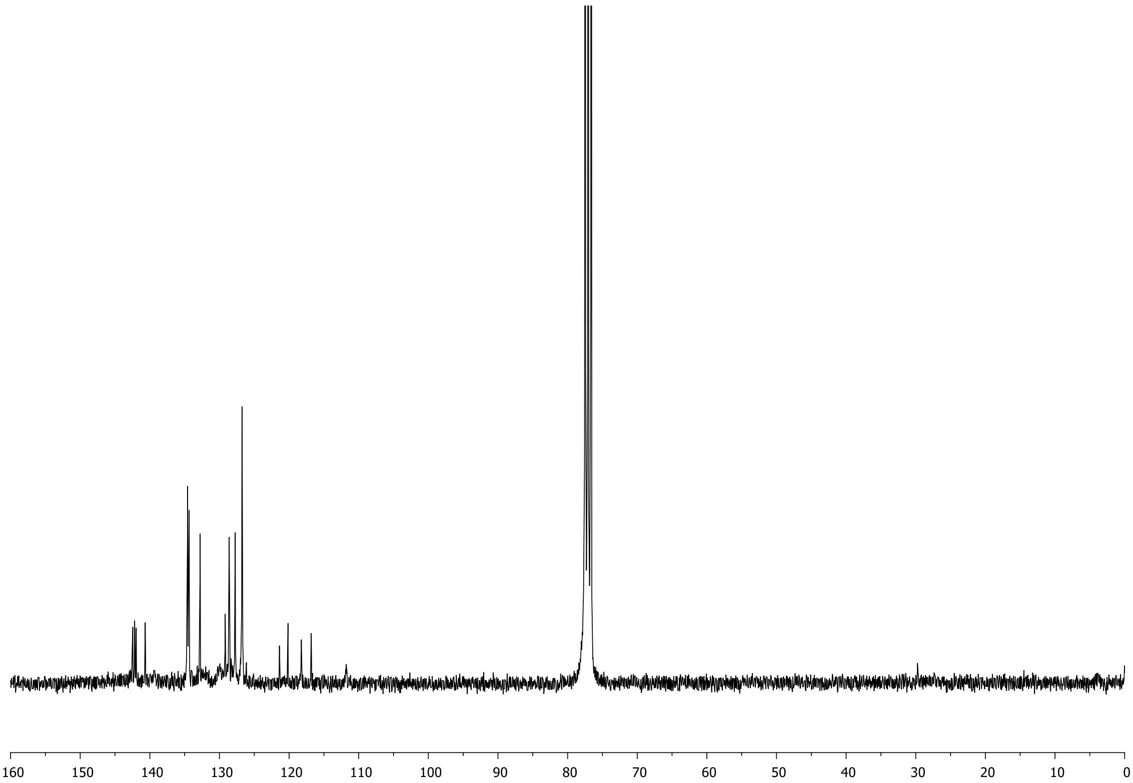

Figure S8. ^{13}C NMR spectrum of **2b** in $CDCl_3$ (75 MHz).

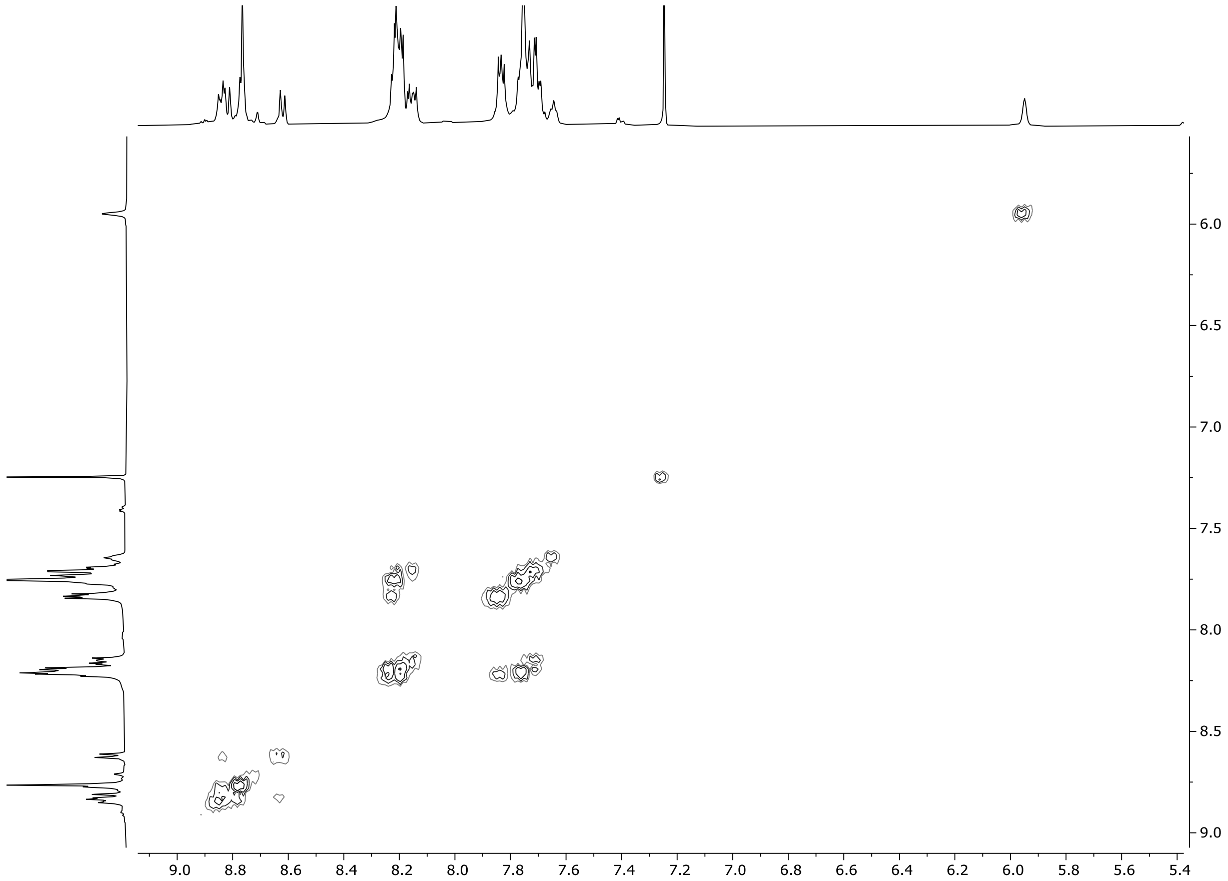

Figure S8.1. ^{1}H/^{1}H COSY spectrum of **2b** in $CDCl_3$.

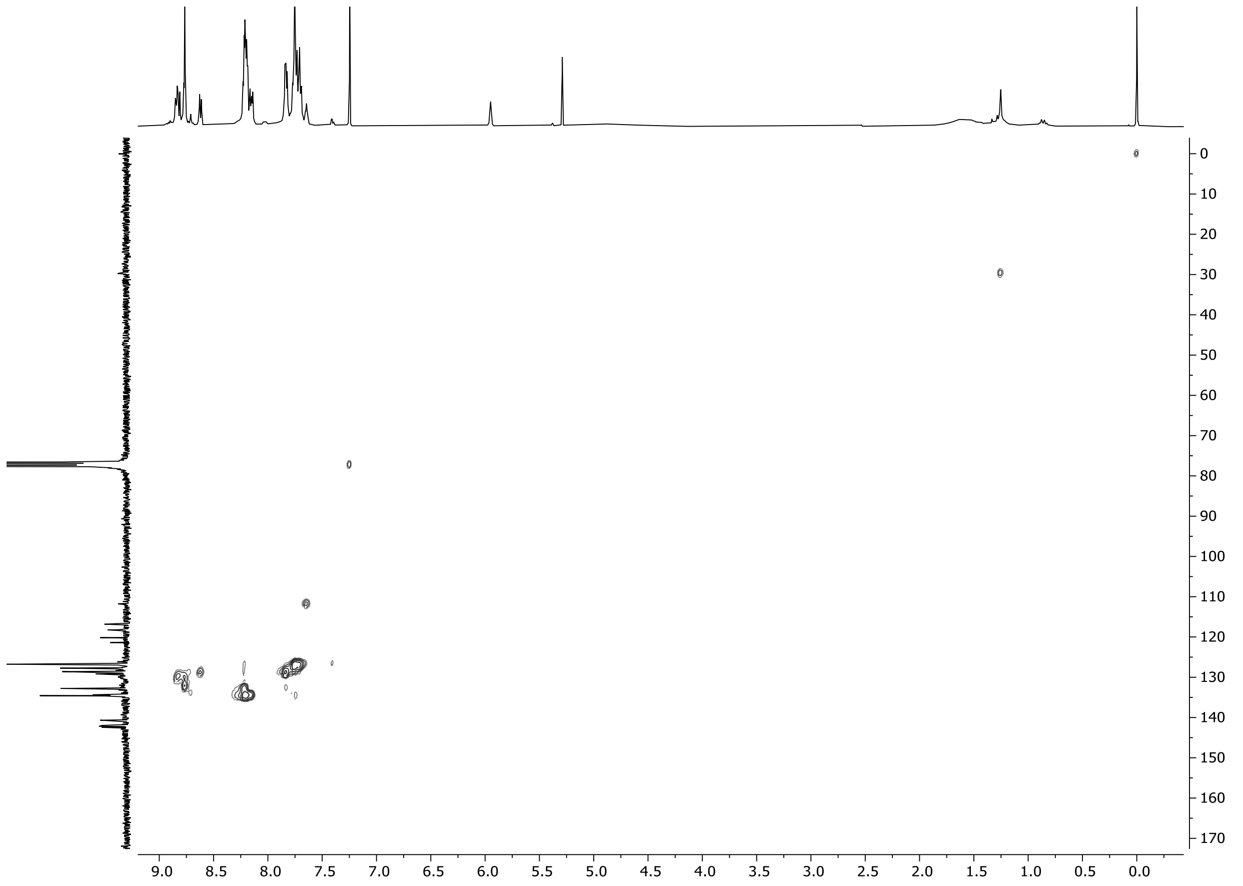

Figure S8.2. $^{1}H/^{13}C$ HSQC spectrum of **2b** in $CDCl_3$.

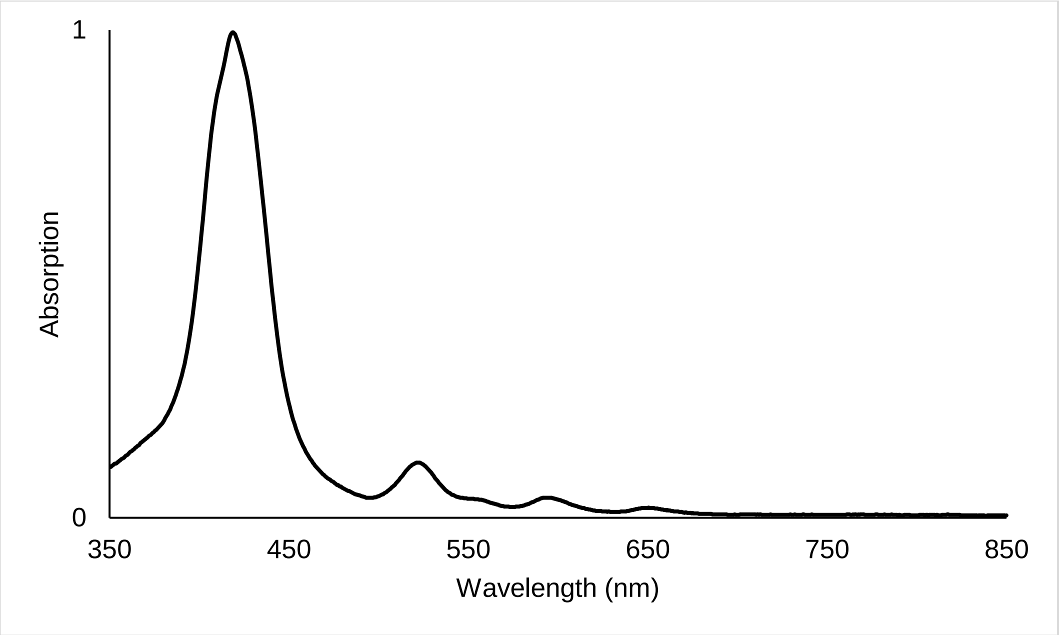

Figure S9. Absorption spectrum of compound **2b** in $CHCl_3$ ($8.5x10^{-6}$ mol.L^{-1}).

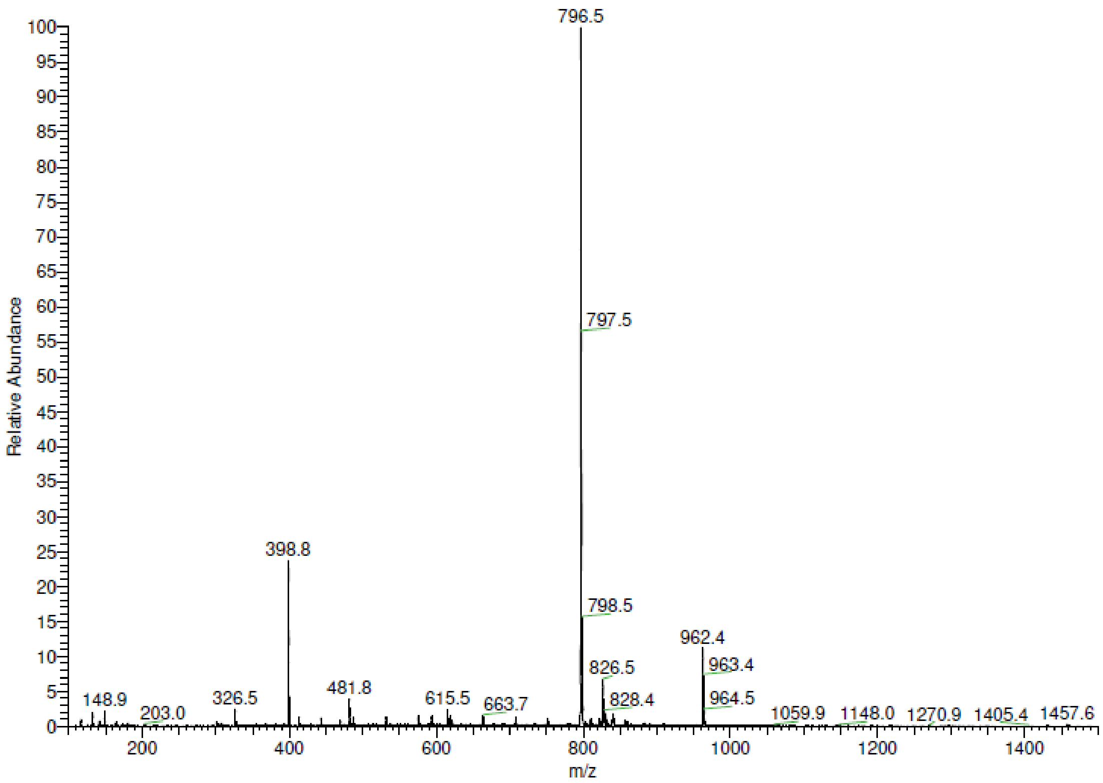

Figure S10. MS-ESI(+) spectrum of **2b**.

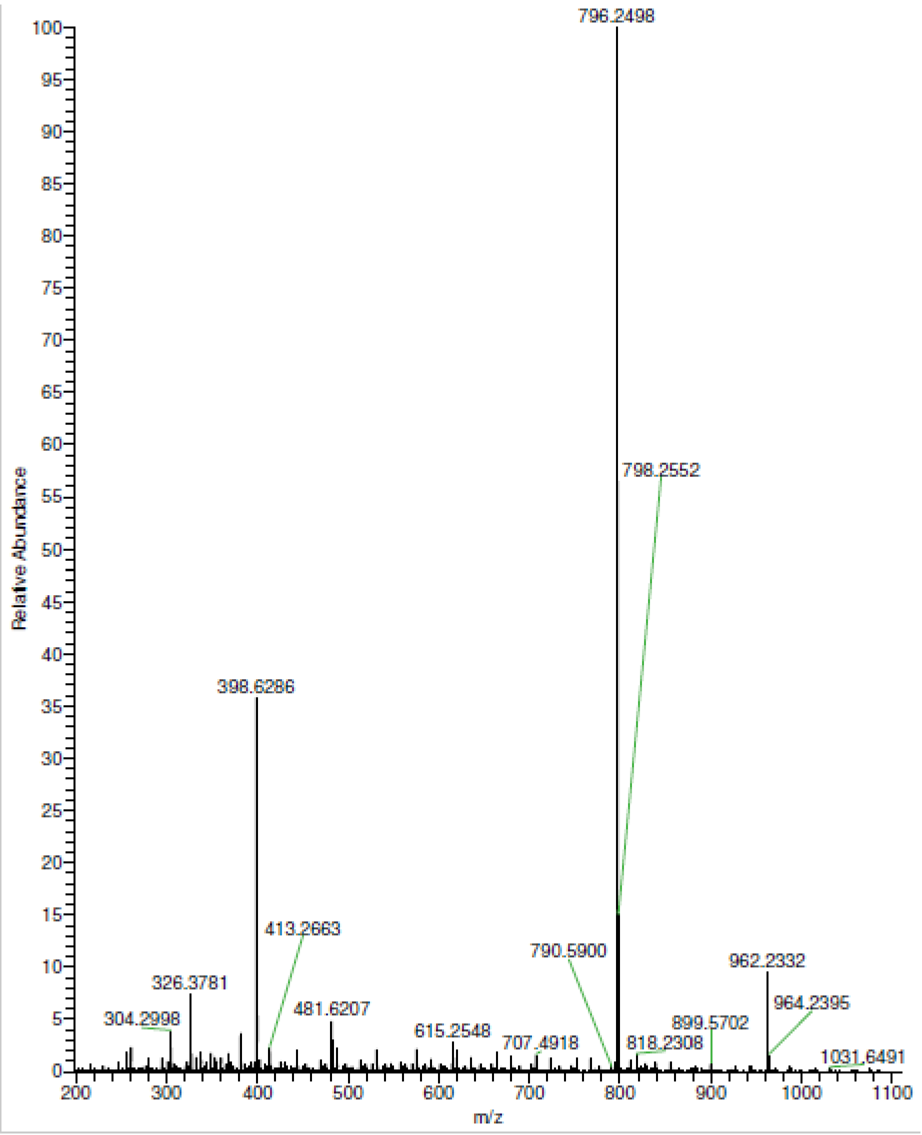

Figure S11. HRMS-ESI(+) of **2b**.

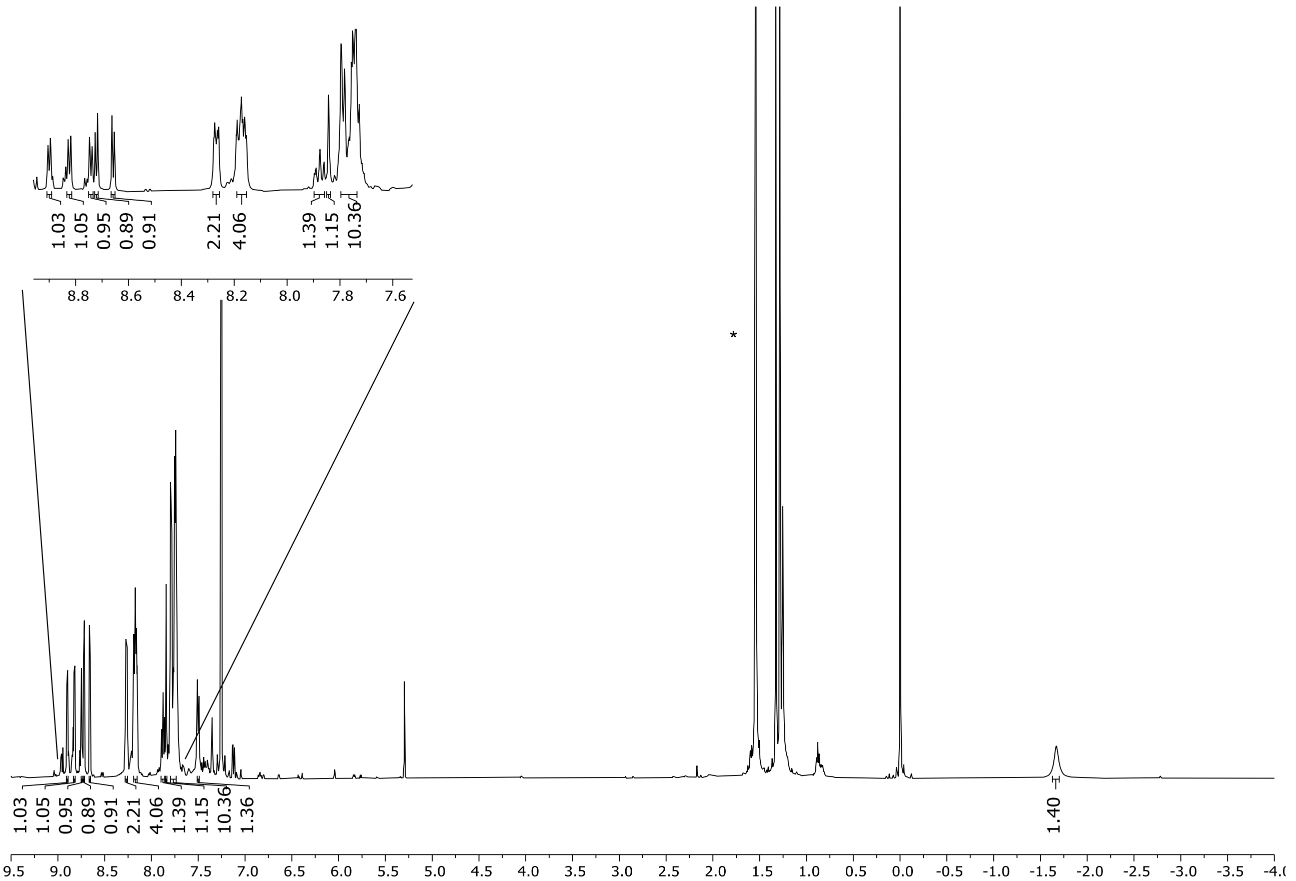

Figure S12. ^{1}H NMR spectrum of **4** in $CDCl_3$ (500 MHz). * Solvents/impurities

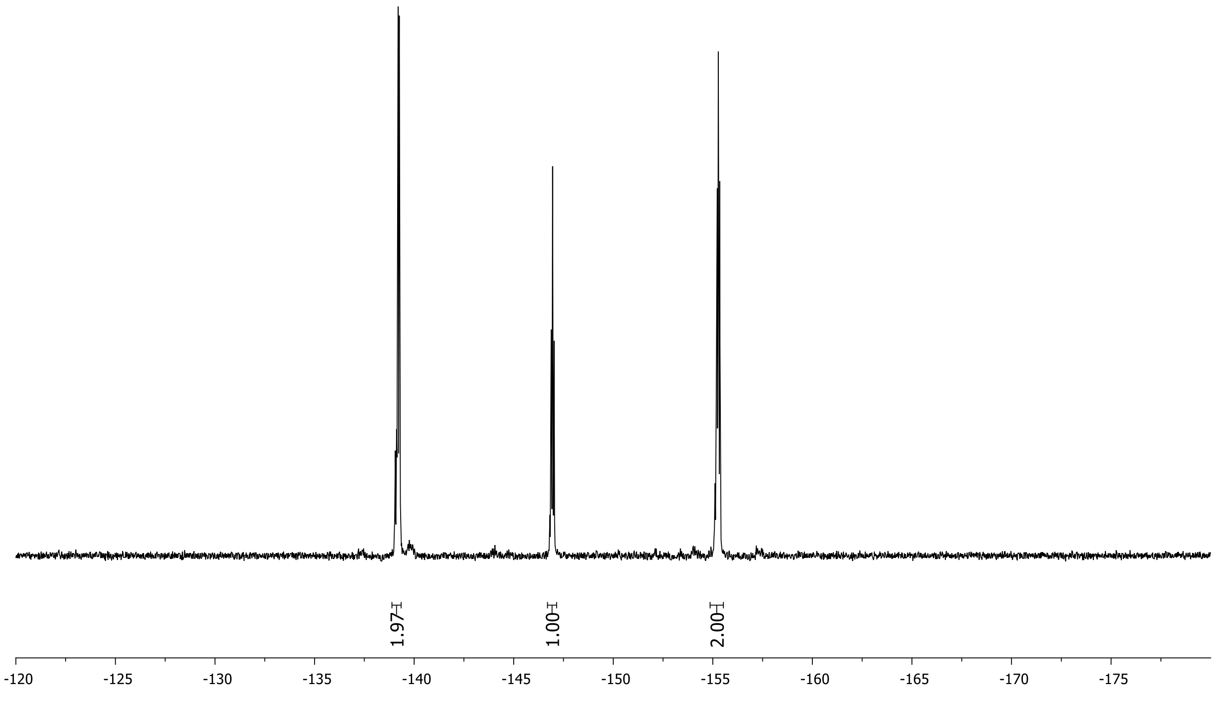

Figure S13. ^{19}F NMR spectrum of compound **4** in $CDCl_3$ (282 MHz).

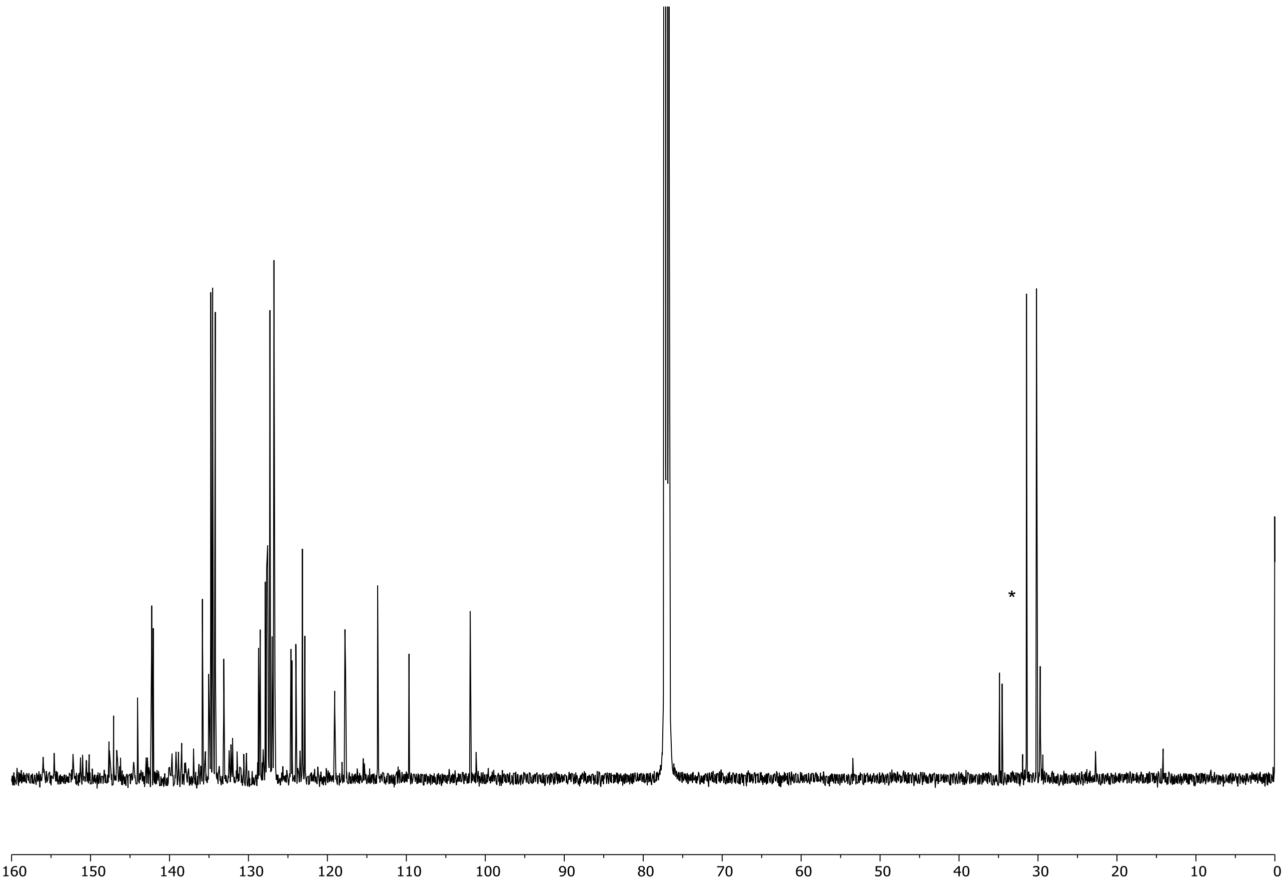

Figure S14. ^{13}C NMR spectrum of **4** in $CDCl_3$ (125 MHz). * Solvents/impurities

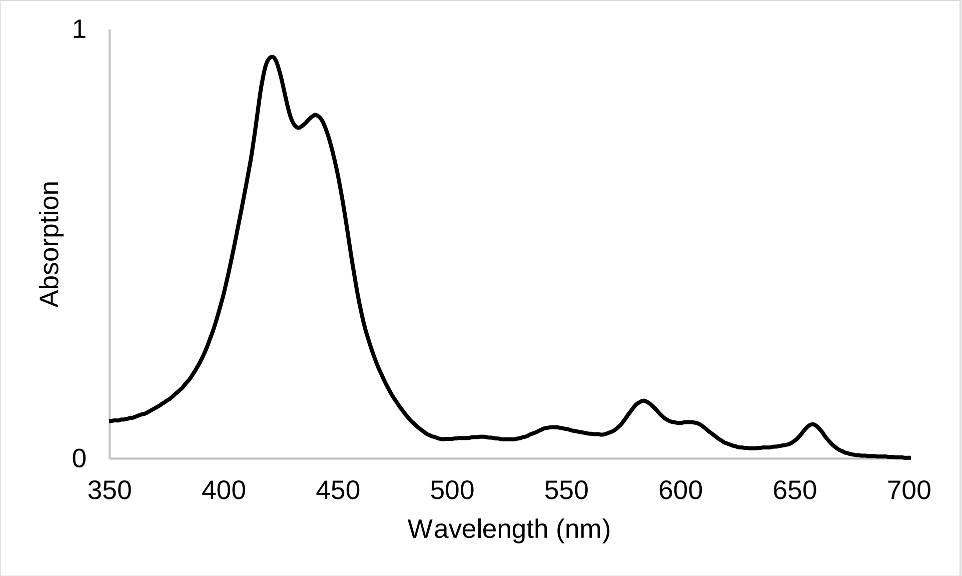

Figure S15. Absorption spectrum of compound **4** in $CHCl_3$ ($5.1x10^{-6}$ $mol.L^{-1}$).

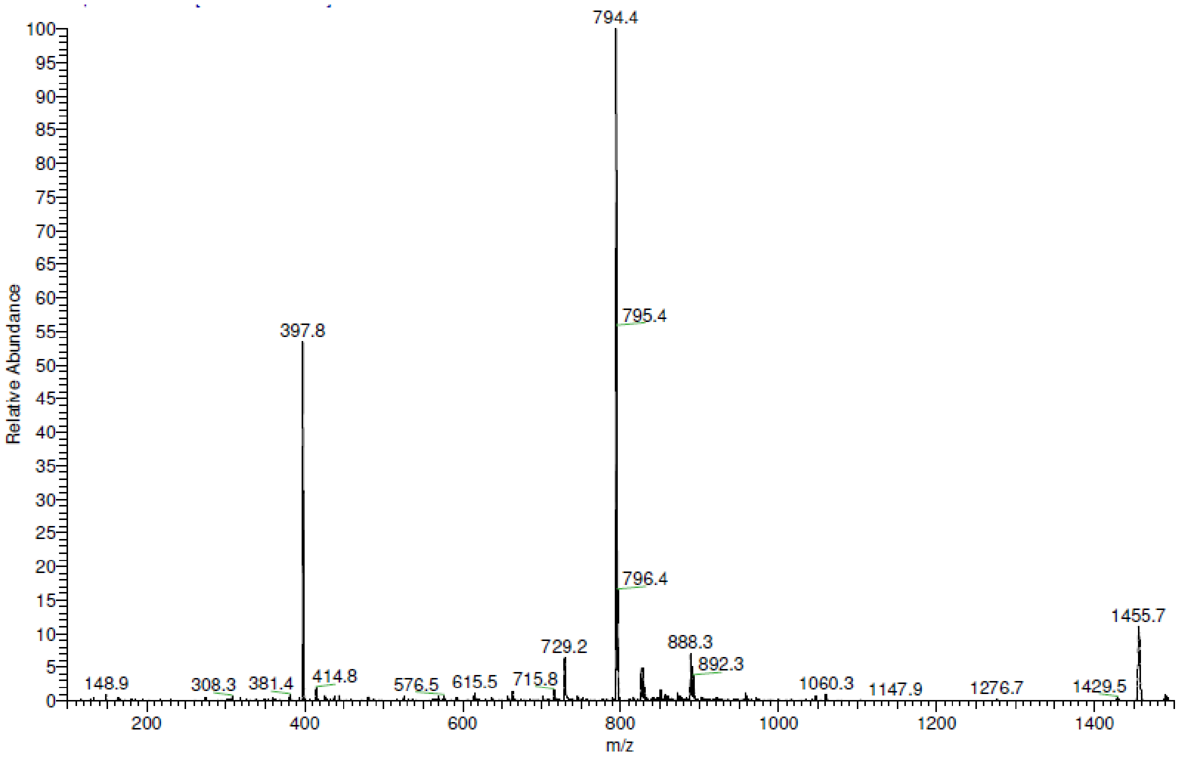

Figure S16. MS-ESI(+) spectrum of **4**.

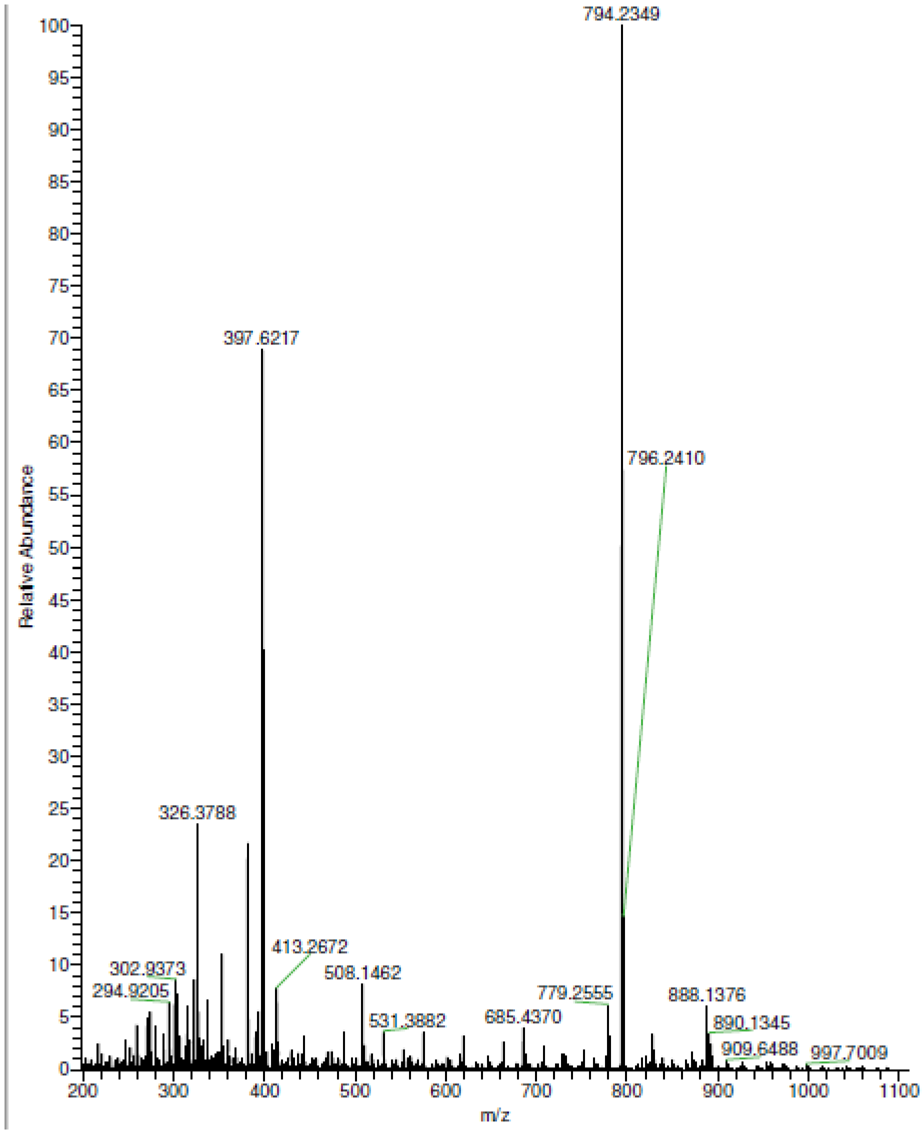

Figure S17. HRMS-ESI(+) of **4**.

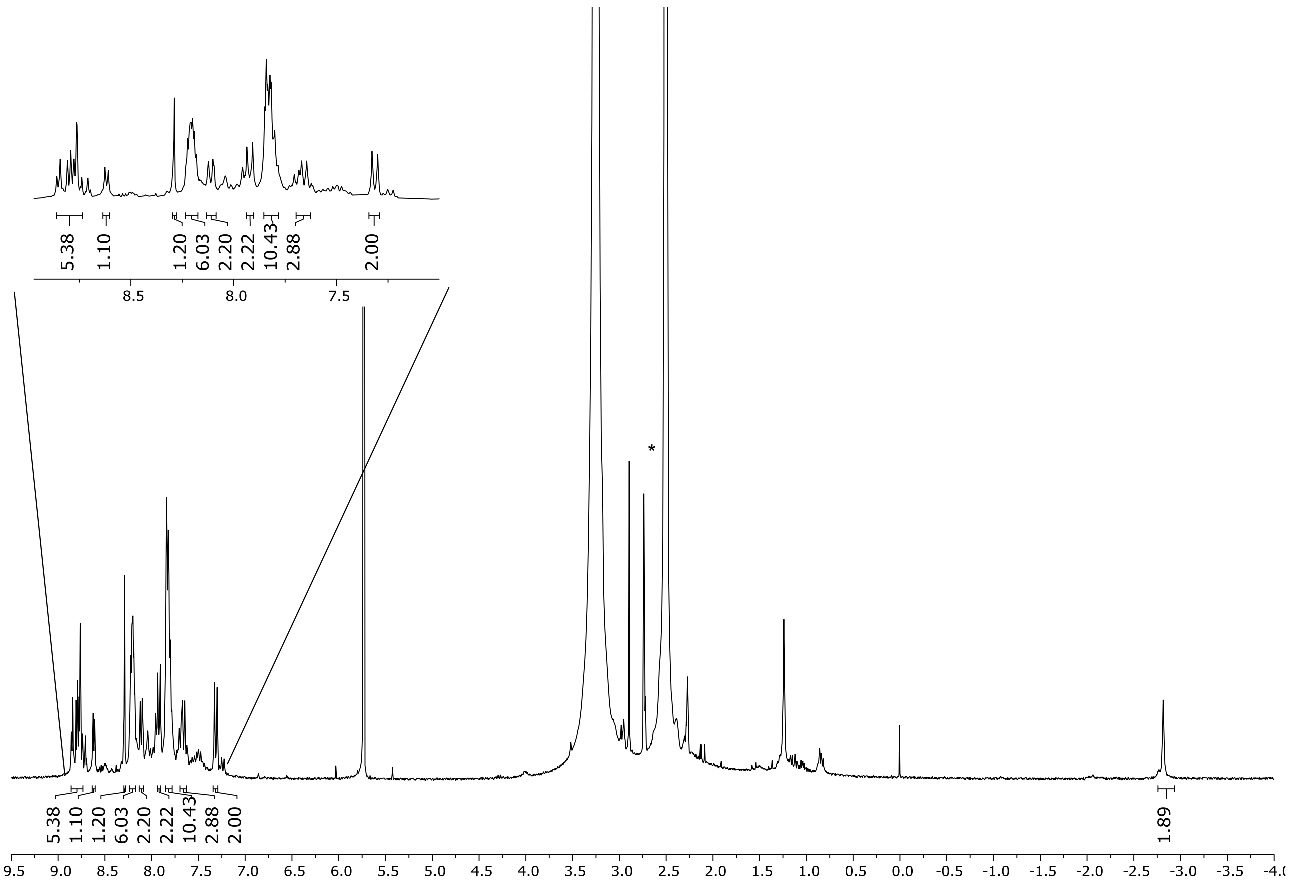

Figure S18. ^{1}H NMR spectrum of **5a** in DMSO-d_6 (300 MHz, 40 °C). * Solvents/impurities

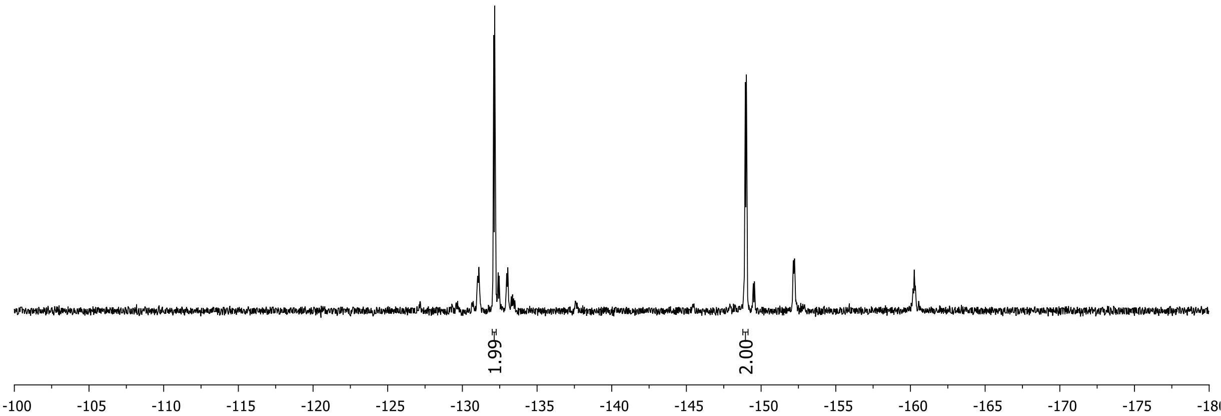

Figure S19. ^{19}F NMR spectrum of compound **5a** in DMSO-d_6 (282 MHz).

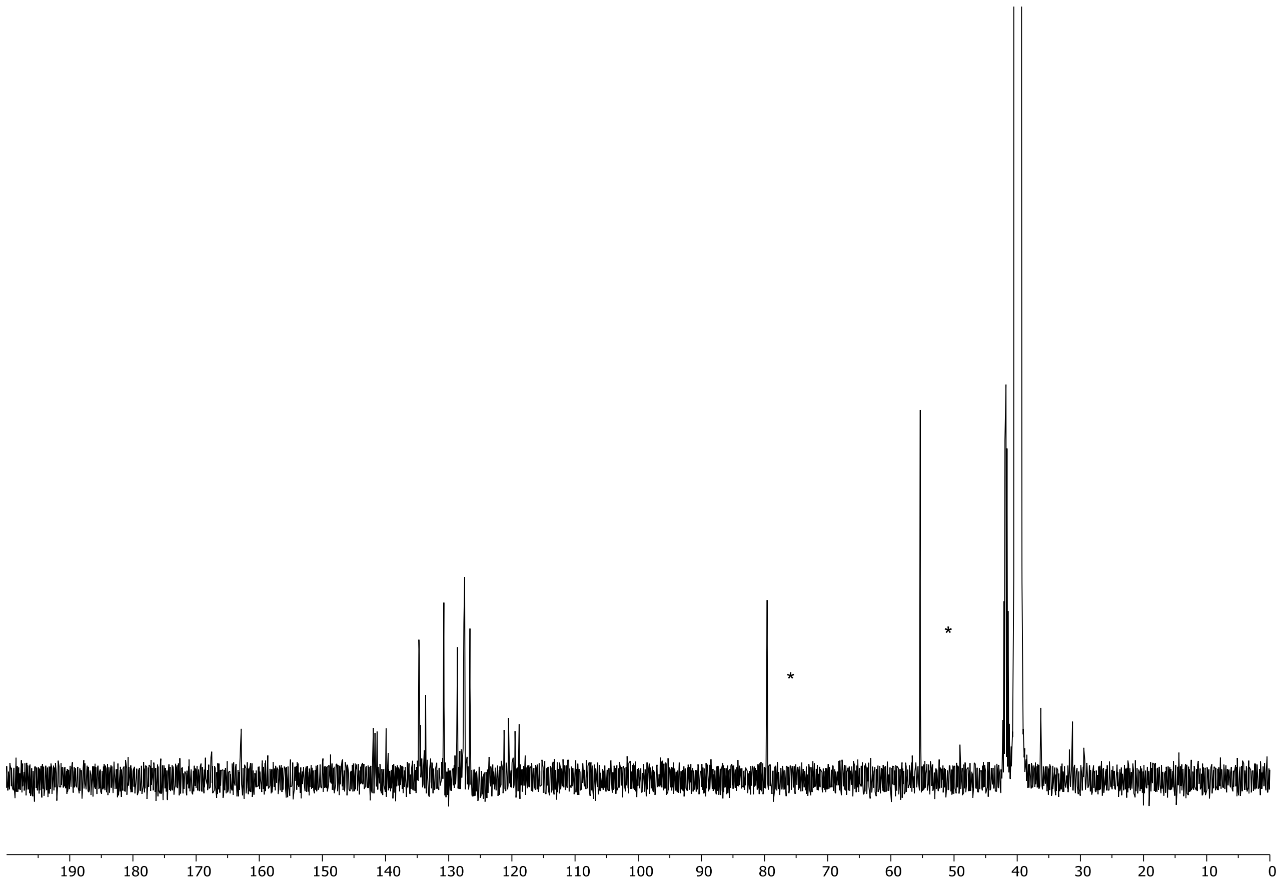

Figure S20. ^{13}C NMR spectrum of **5a** in DMSO-d_6 (125 MHz). * Solvents/impurities

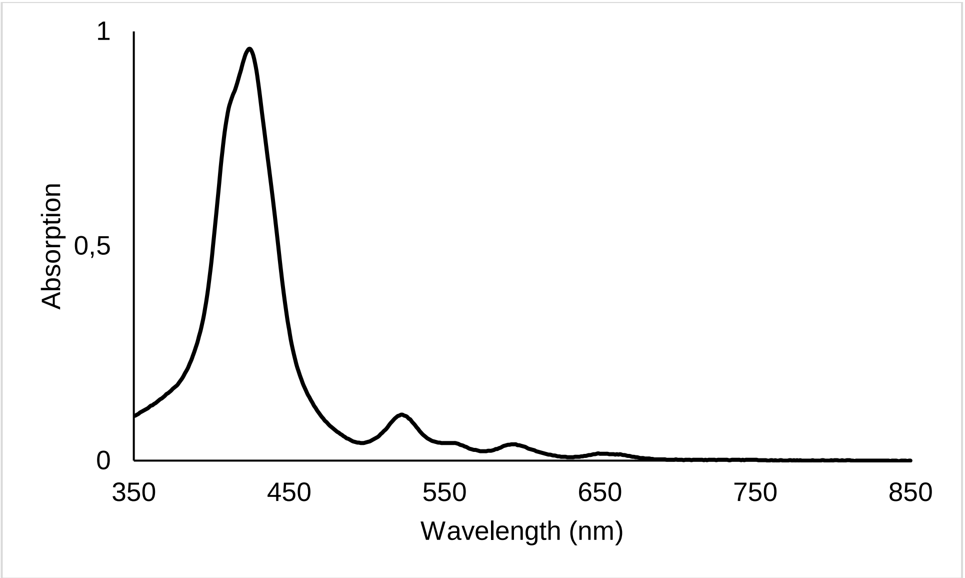

Figure S21. UV-Vis absorption spectrum of compound **5a** in $CHCl_3$ ($5.3x10^{-6}$ mol.L^{-1}).

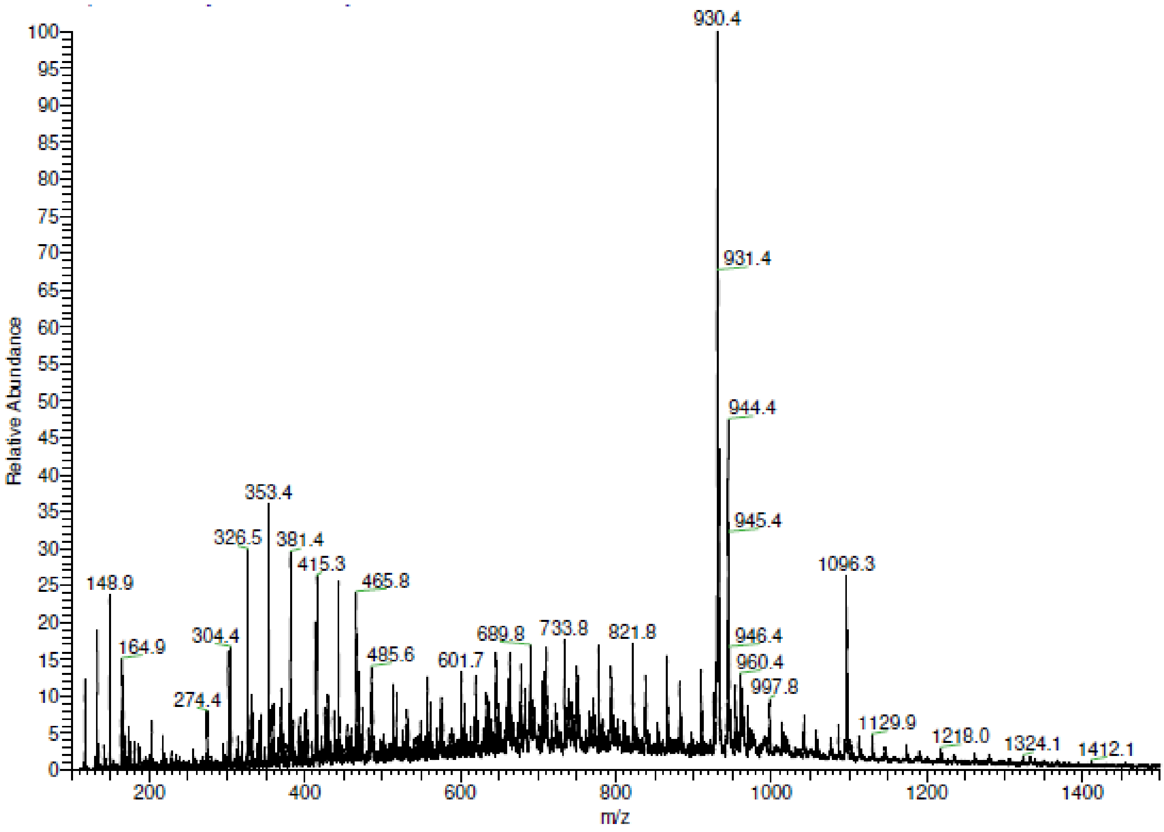

Figure S22. MS-ESI(+) spectrum of **5a**.

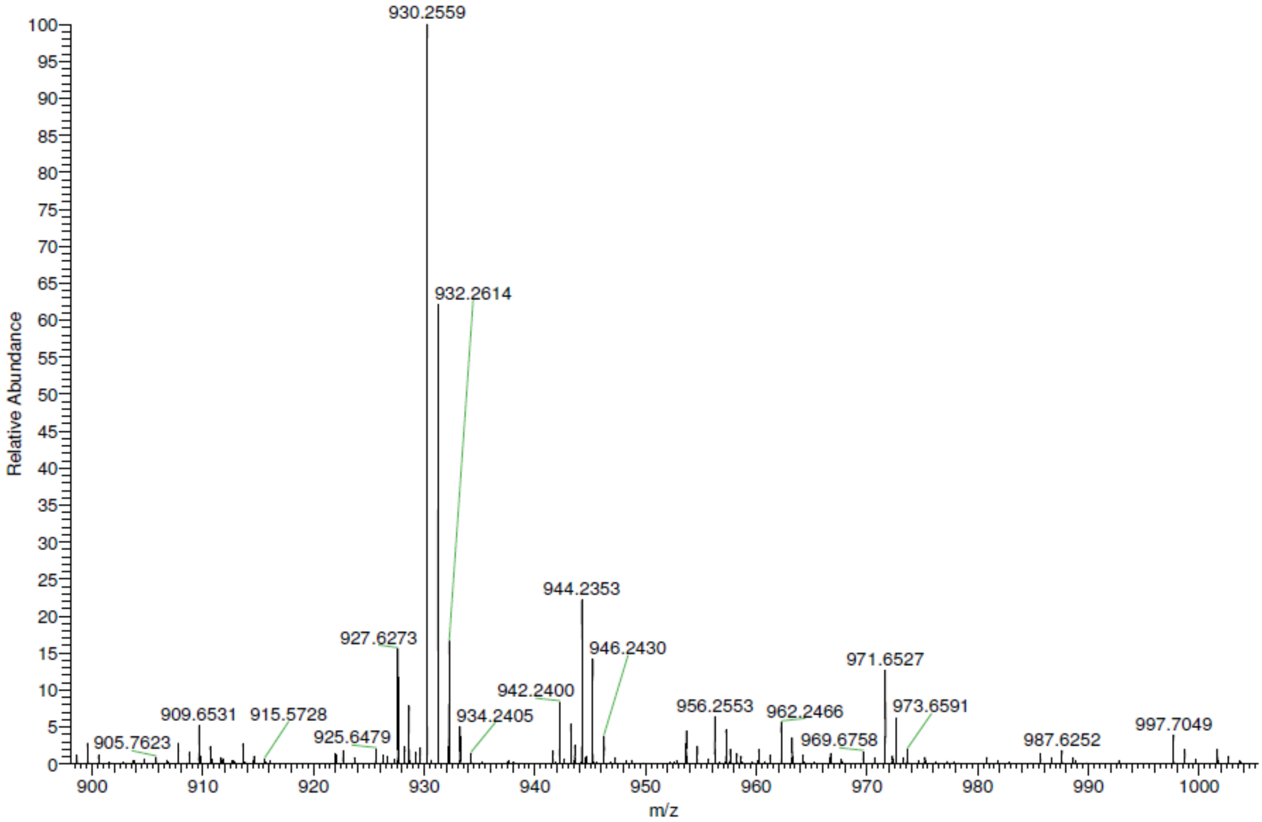

Figure S23. HRMS-ESI(+) of **5a**.

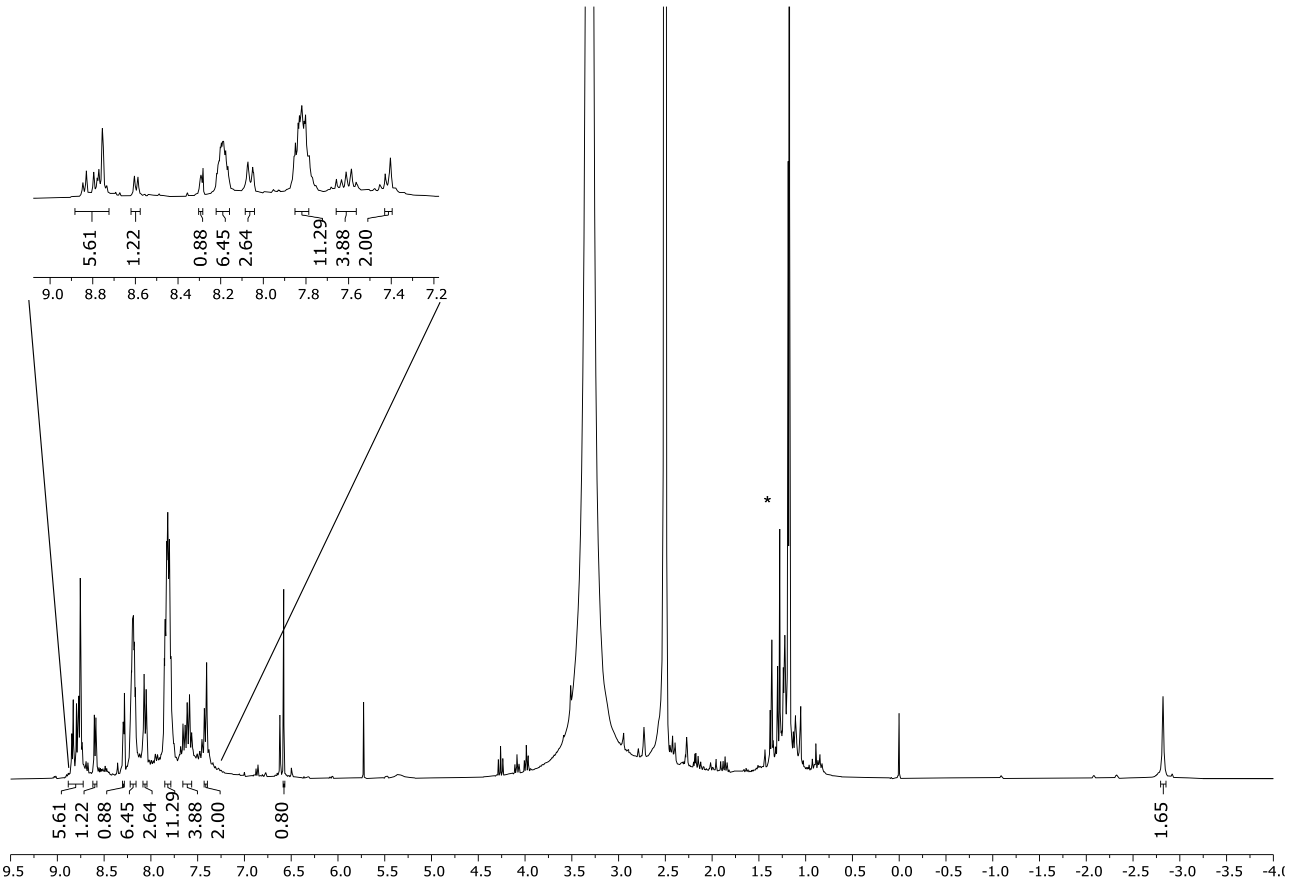

Figure S24. ^{1}H NMR spectrum of **5b** in DMSO-d_6 (300 MHz, 40 °C). * Solvents/impurities

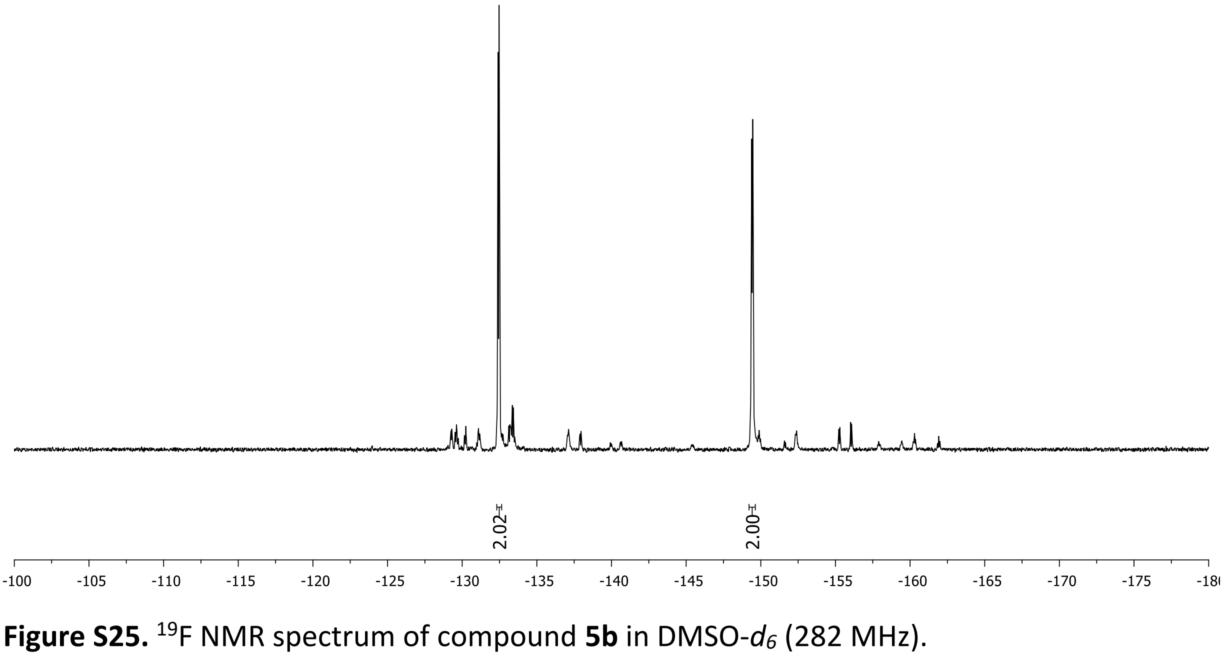

Figure S25. ^{19}F NMR spectrum of compound **5b** in DMSO-d_6 (282 MHz).

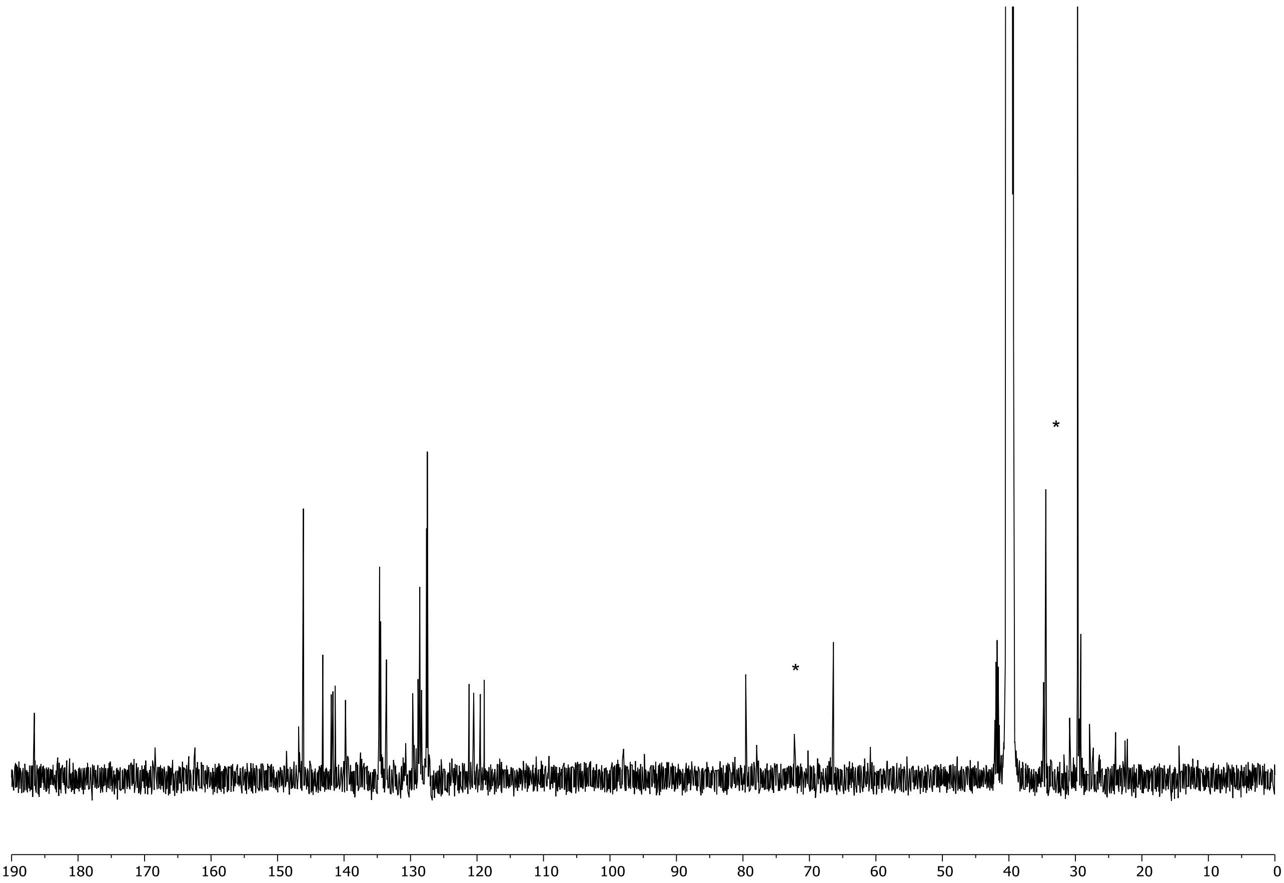

Figure S26. ^{13}C NMR spectrum of **5b** in DMSO-d_6 (125 MHz). * Solvents/impurities

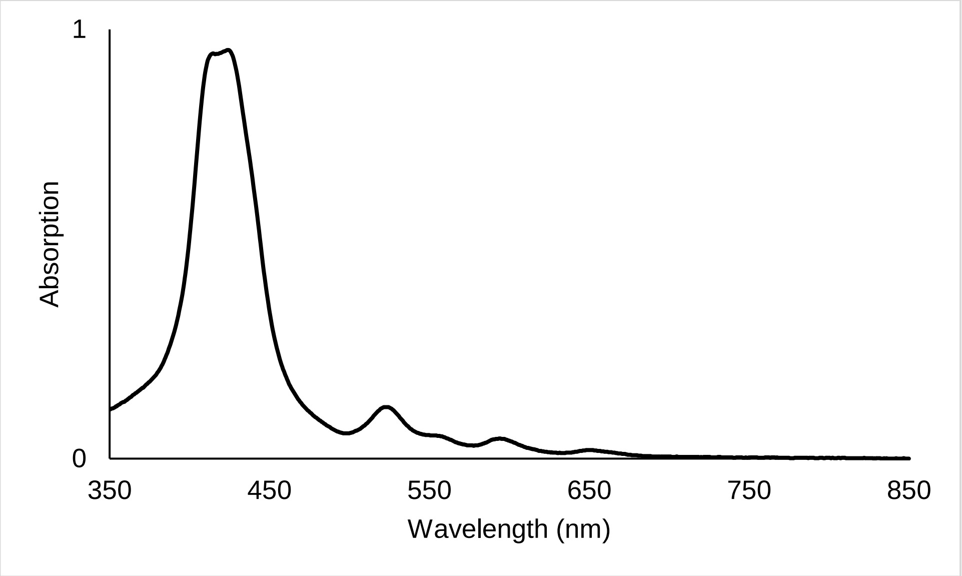

Figure S27. Absorption spectrum of compound **5b** in $CHCl_3$ (8.9×10^{-6} mol.L^{-1}).

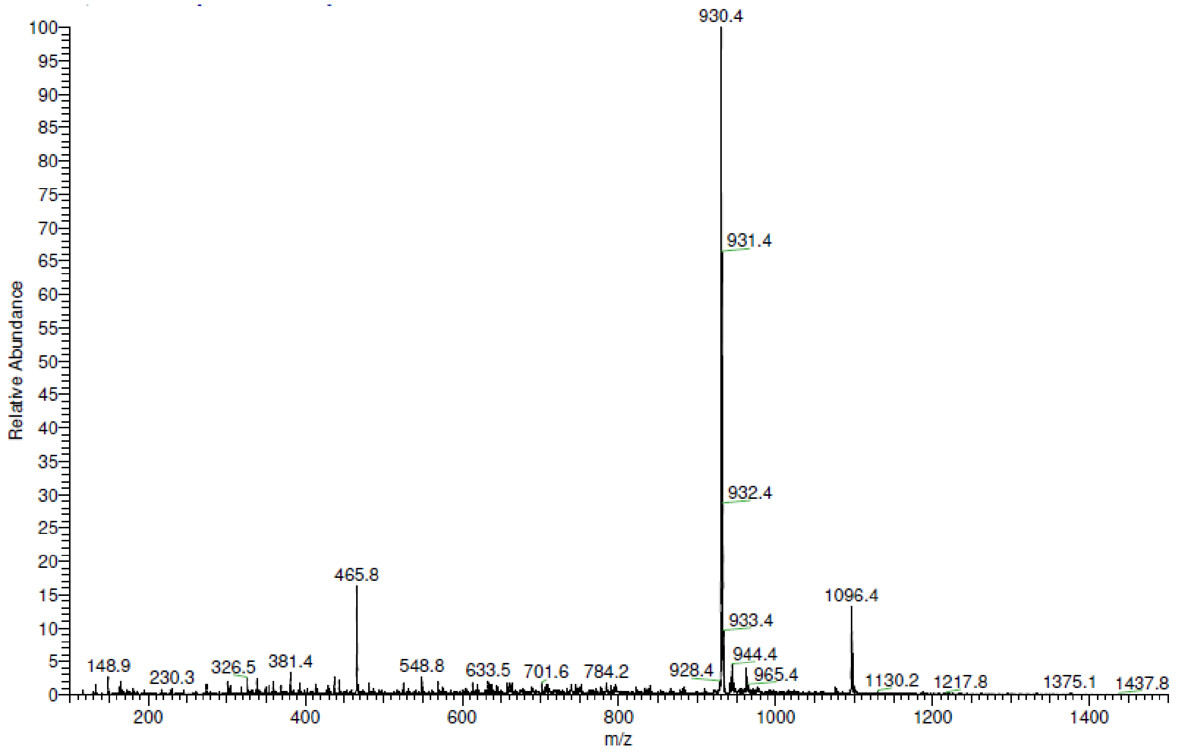

Figure S28. MS-ESI(+) spectrum of **5b**.

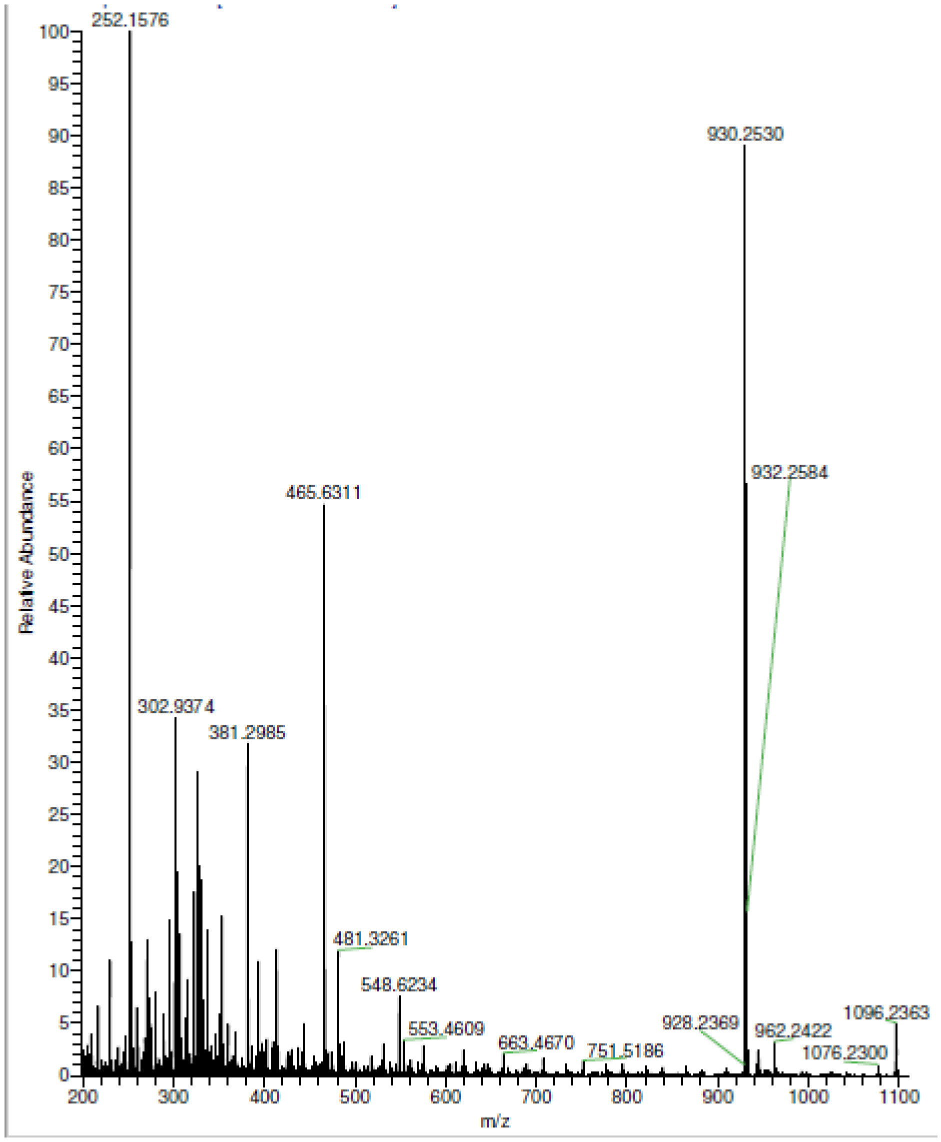

Figure S29. HRMS-ESI(+) of **5b**.

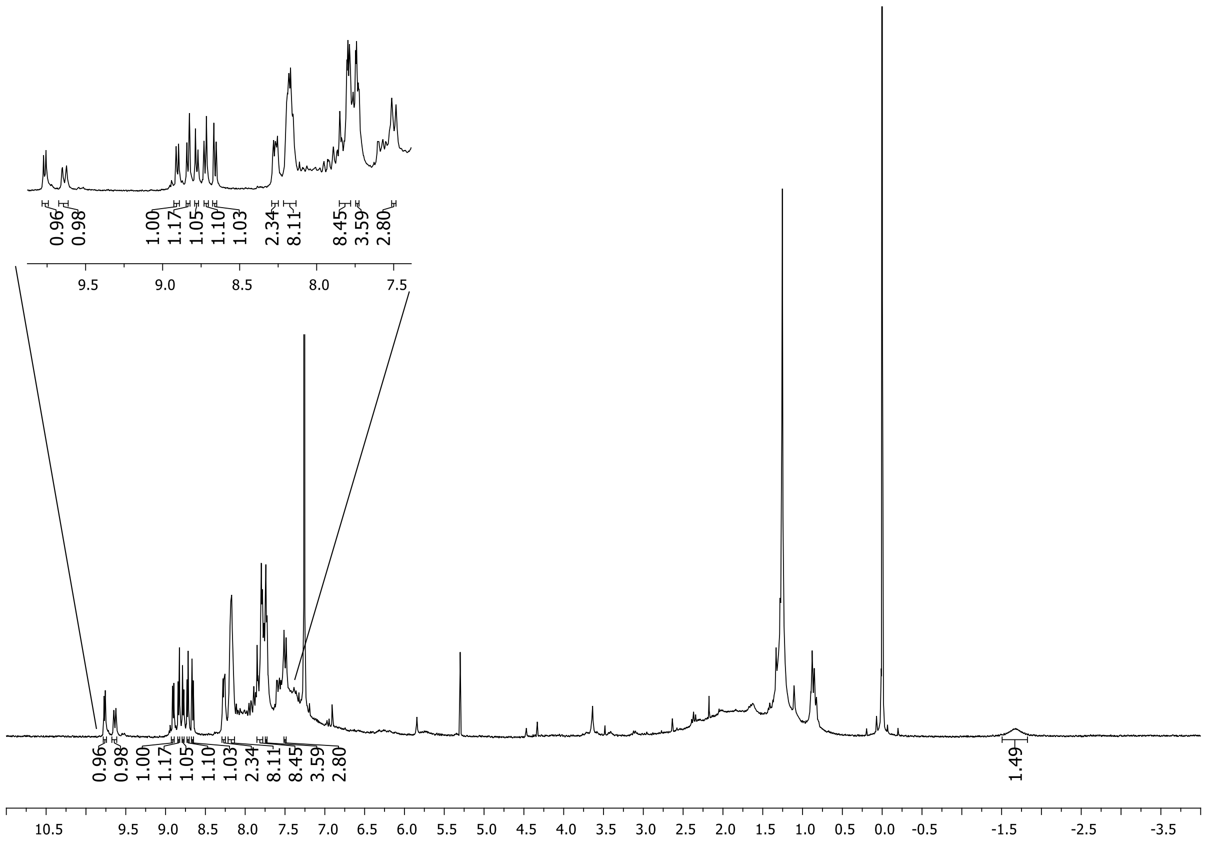

Figure S30. ^{1}H NMR spectrum of **6a** in $CDCl_3$ (300 MHz).

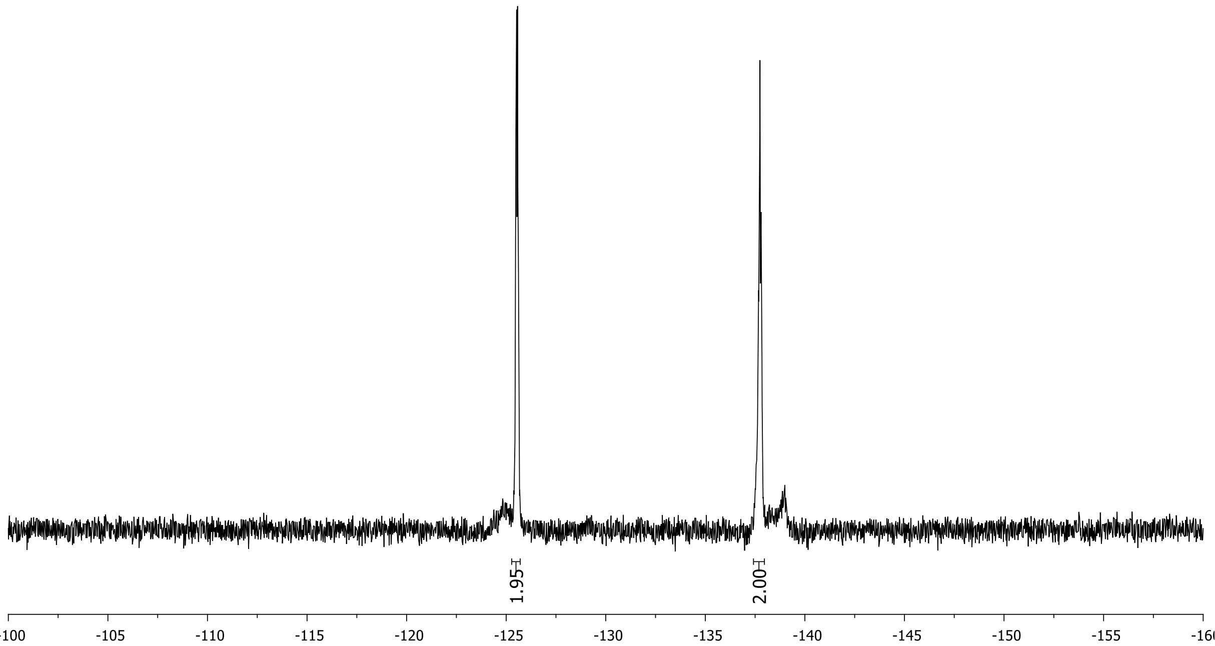

Figure S31. ^{19}F NMR spectrum of compound **6a** in $CDCl_3$ (282 MHz).

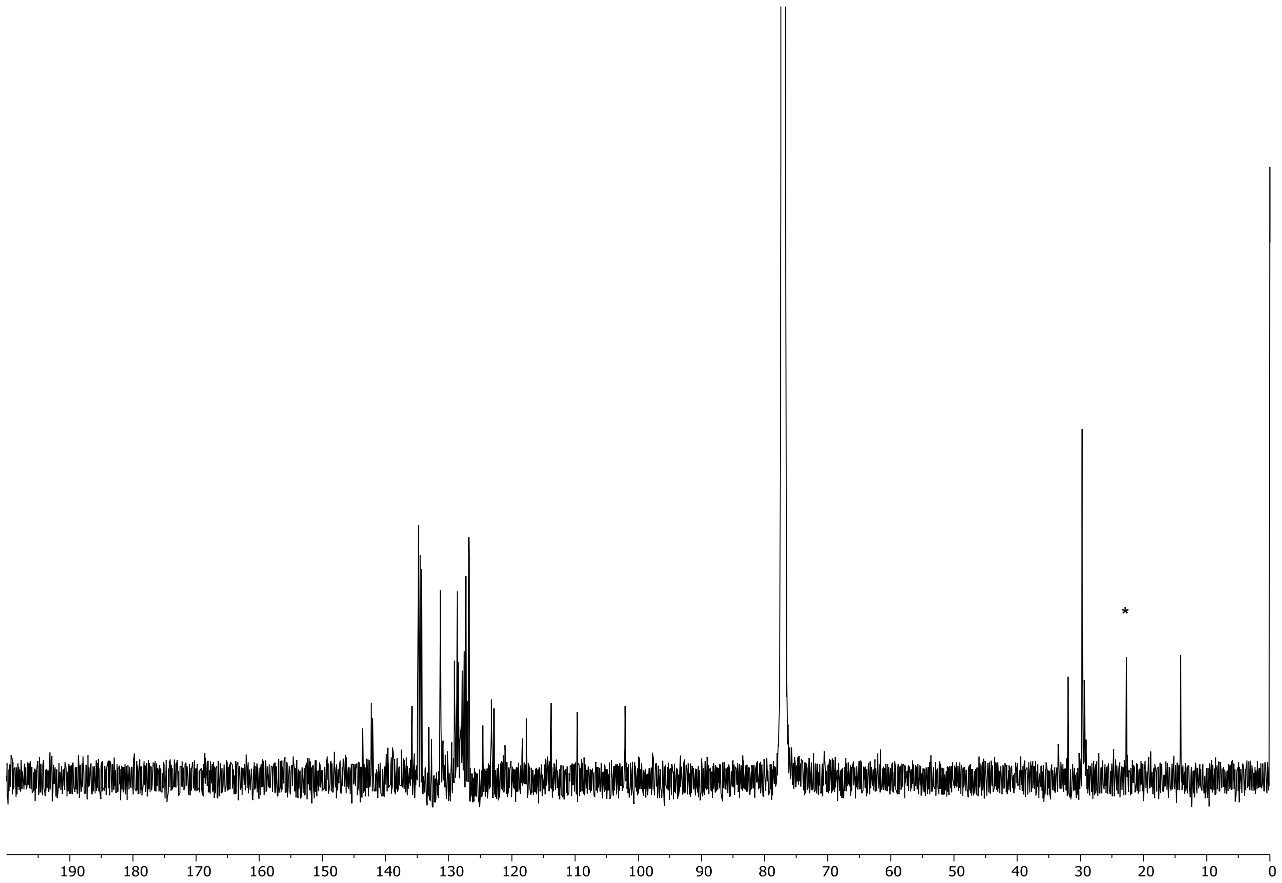

Figure S32. ^{13}C NMR spectrum of **6a** in $CDCl_3$ (125 MHz). * Solvents/impurities

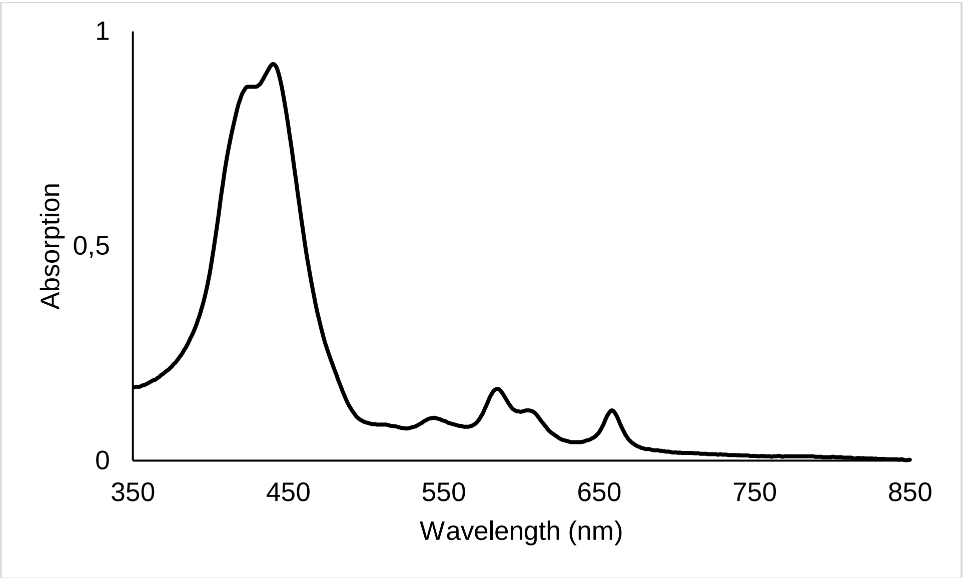

Figure S33. Absorption spectrum of compound **6a** in $CHCl_3$ ($3.0x10^{-5}$ mol.L^{-1}).

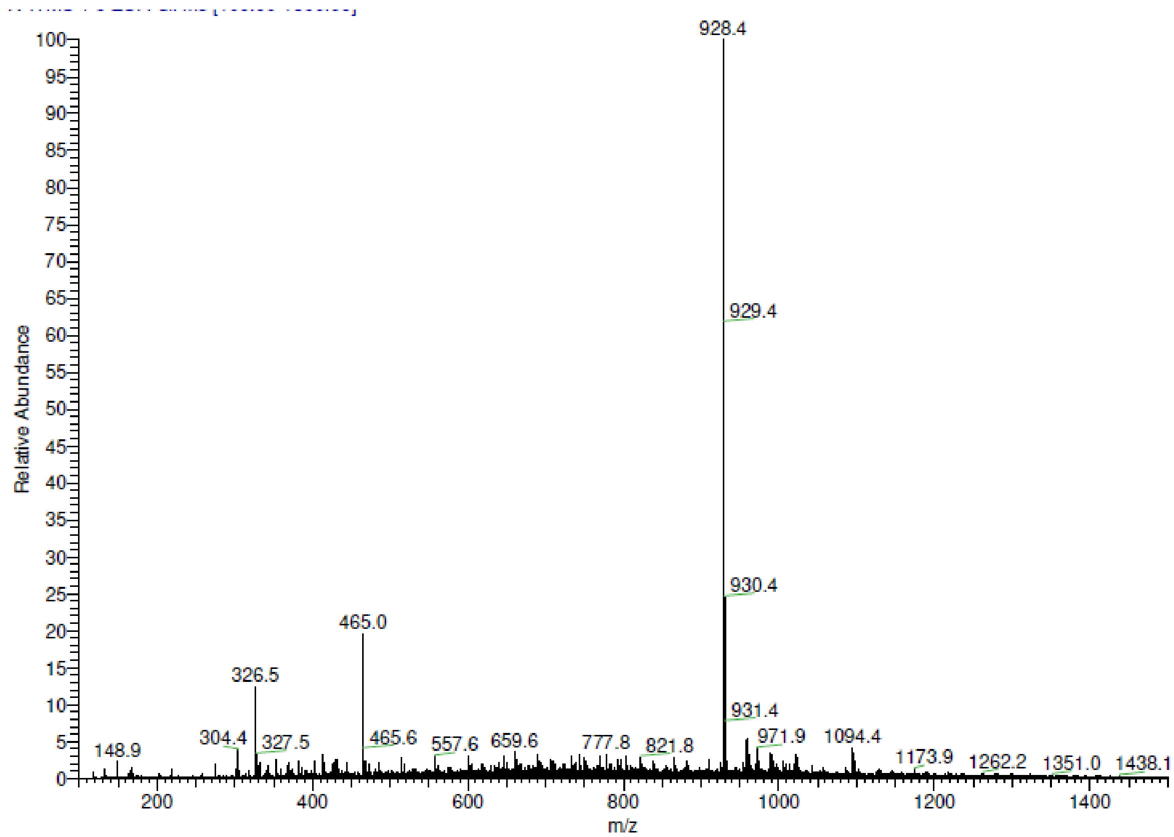

Figure S34. MS-ESI(+) spectrum of **6a**.

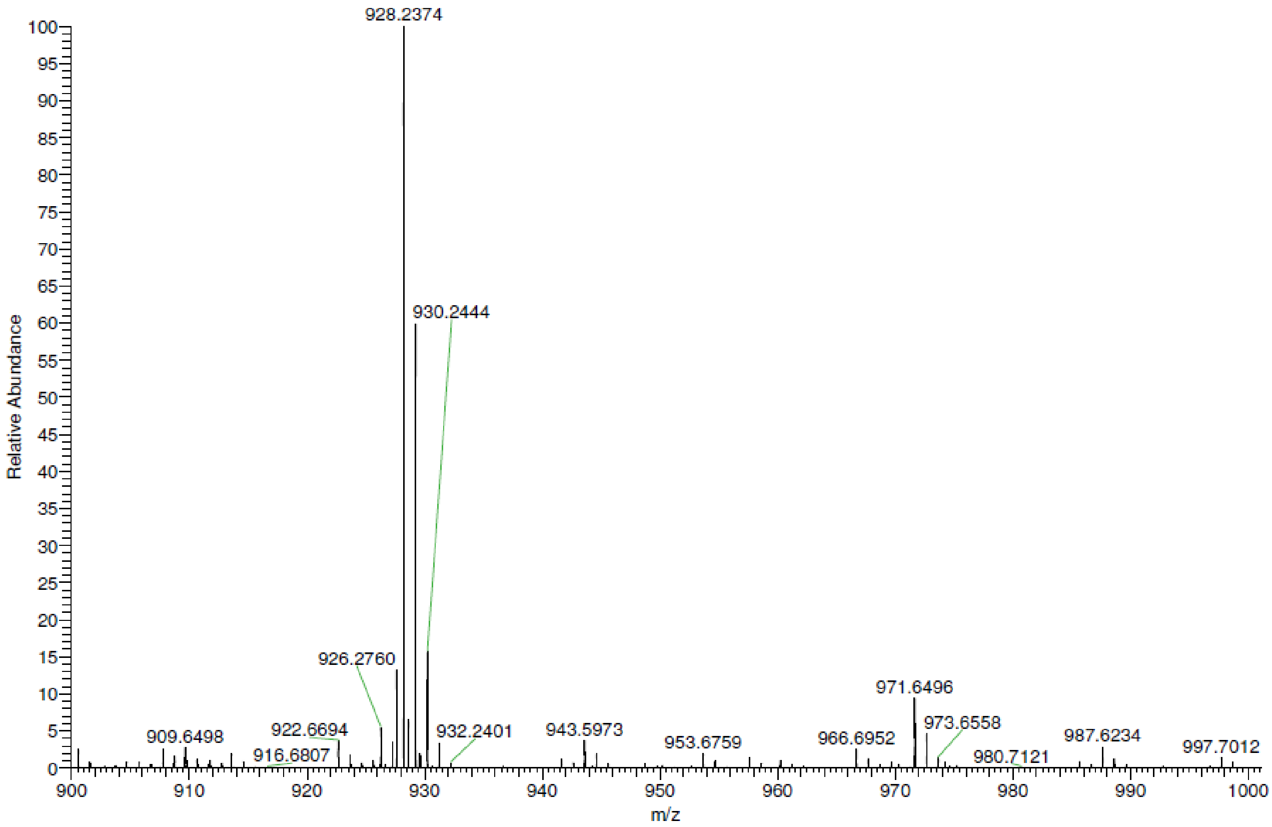

Figure S35. HRMS-ESI(+) of **6a**.

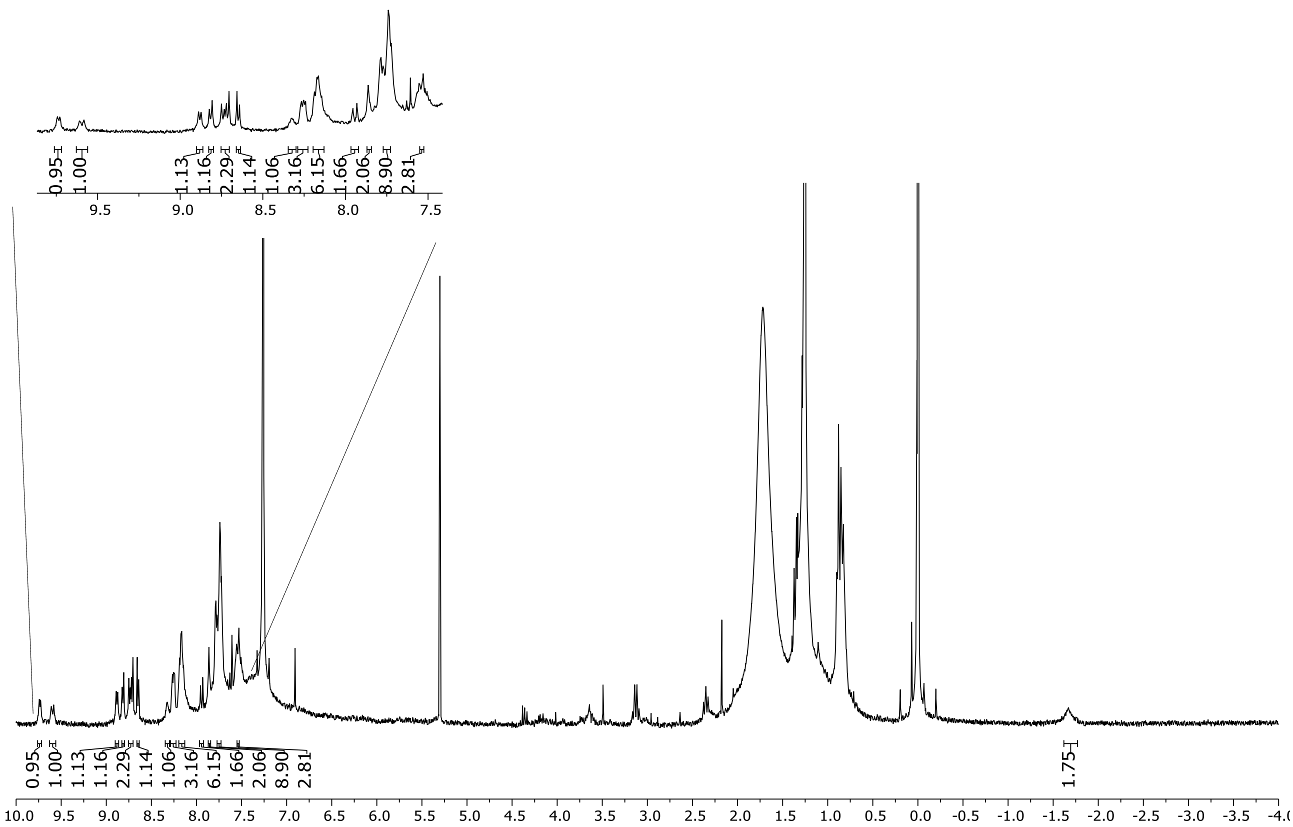

Figure S36. ^{1}H NMR spectrum of compound **6b** in $CDCl_3$ (300 MHz).

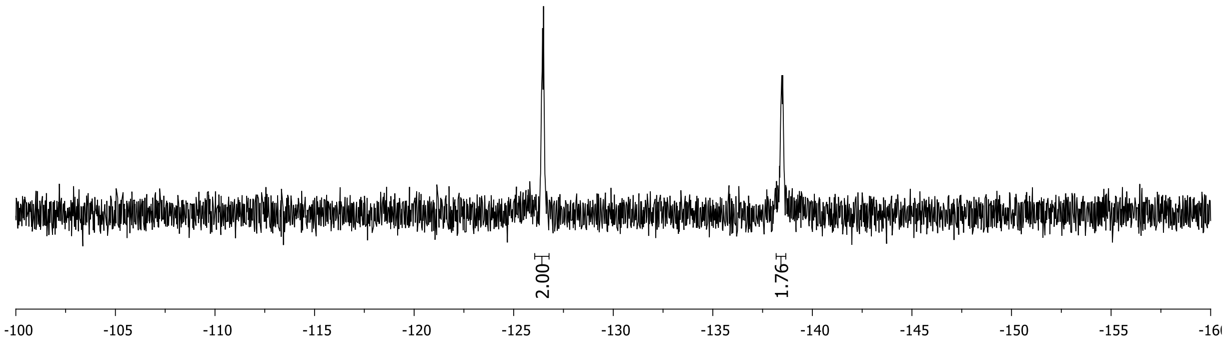

Figure S37. ^{19}F NMR spectrum of compound **6b** in $CDCl_3$ (282 MHz).

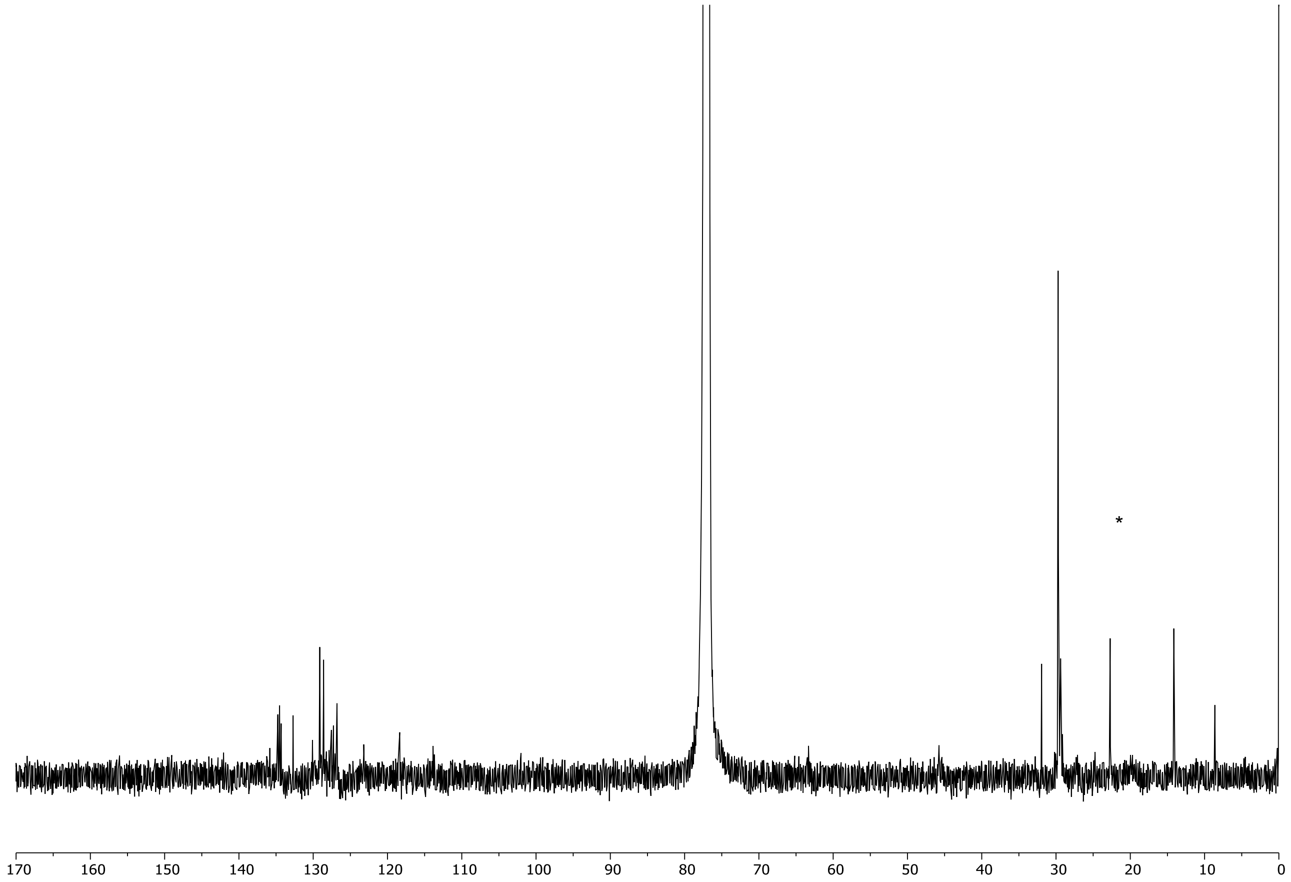

Figure S38. ^{13}C NMR spectrum of **6b** in $CDCl_3$ (125 MHz). * Solvents/impurities

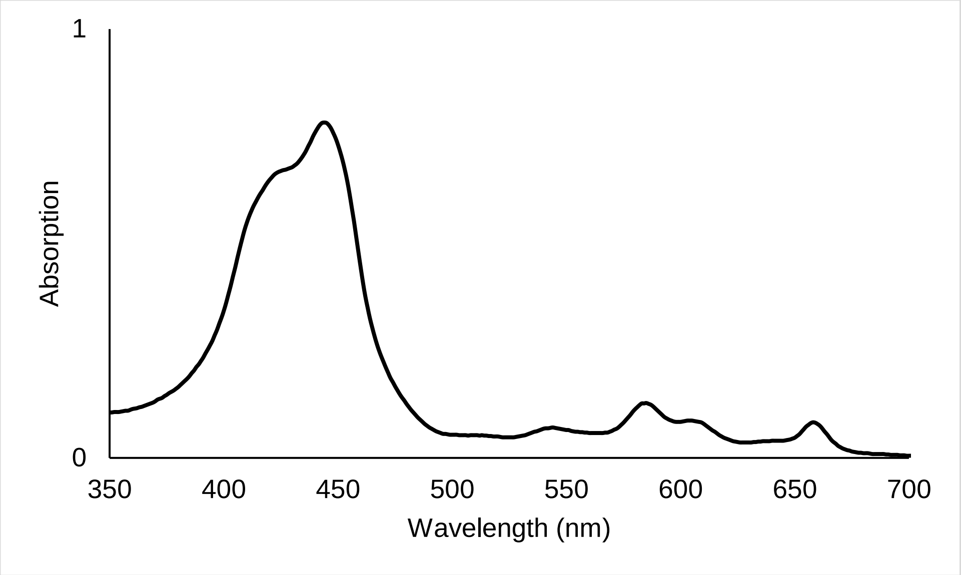

Figure S39. Absorption spectrum of compound **6b** in $CHCl_3$ ($2.5x10^{-5}$ mol.L^{-1}).

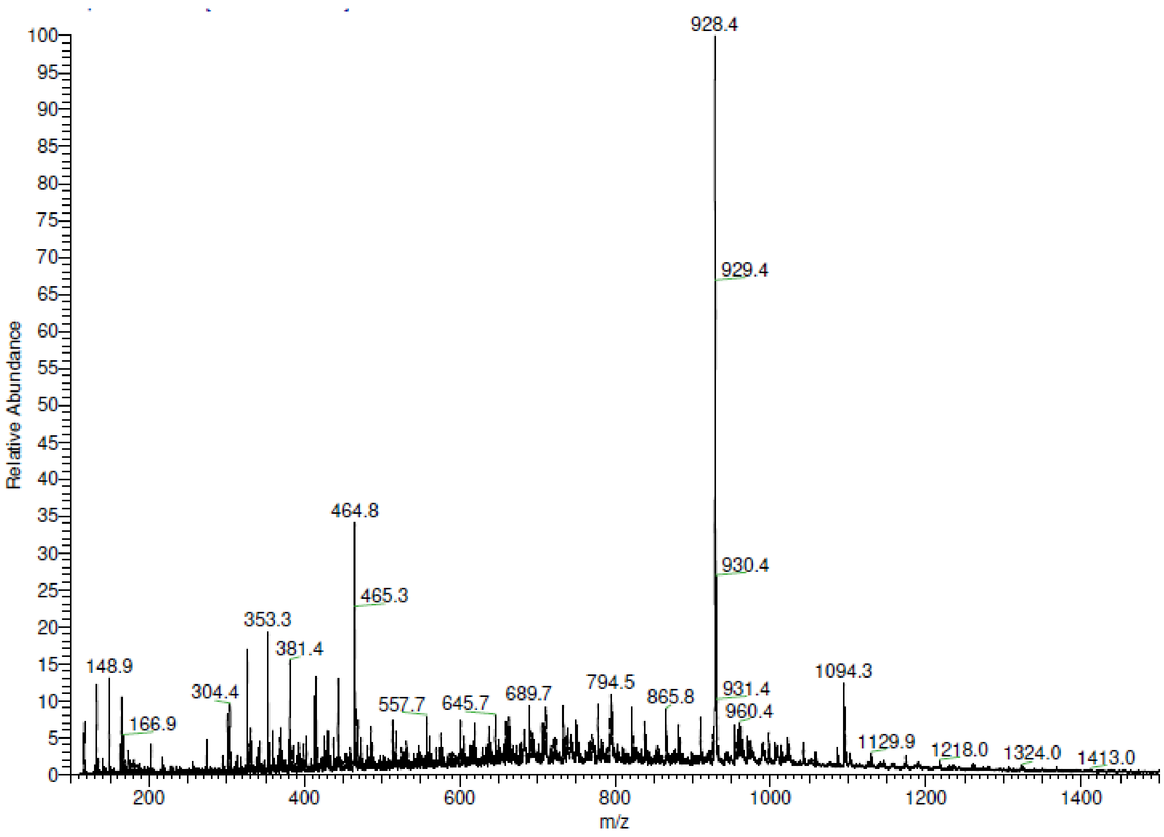

Figure S40. MS-ESI(+) spectrum of **6b**.

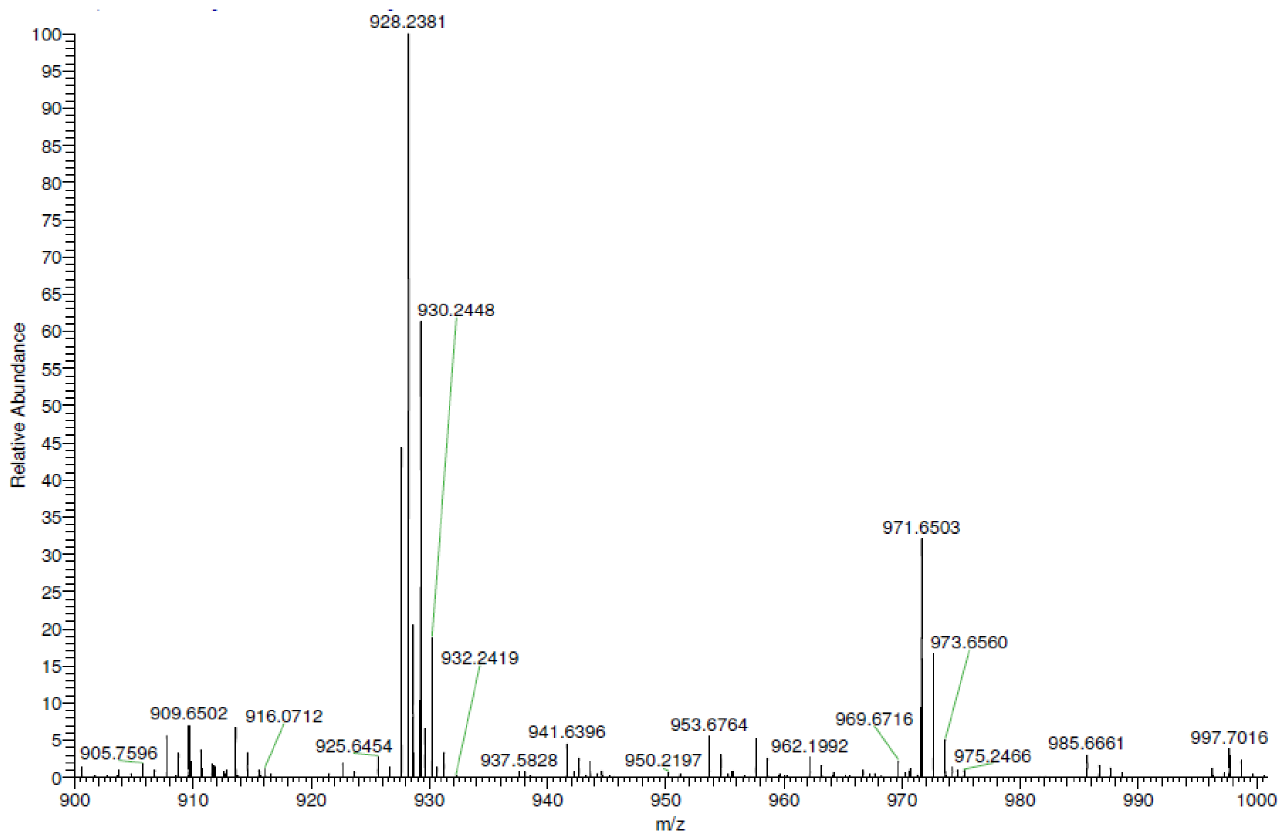

Figure S41. HRMS-ESI(+) of **6b**.

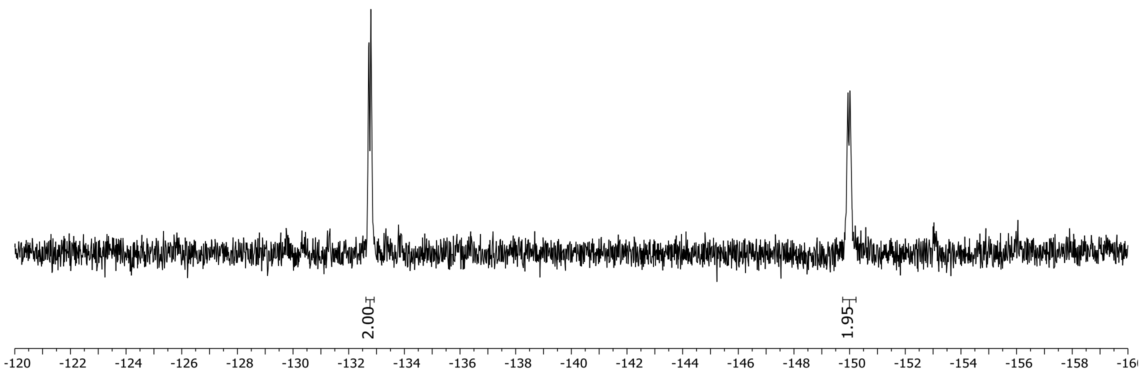

Figure S42. ^{19}F NMR spectrum of compound **5c** in $CDCl_3$ (282 MHz).

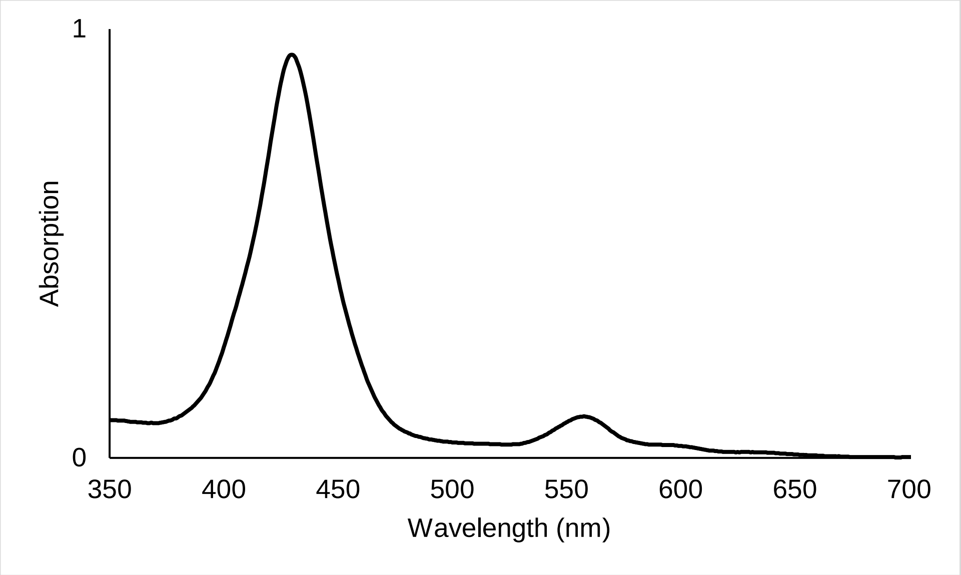

Figure S43. Absorption spectrum of compound **5c** in $CHCl_3$ ($1.6x10^{-5}$ $mol.L^{-1}$).

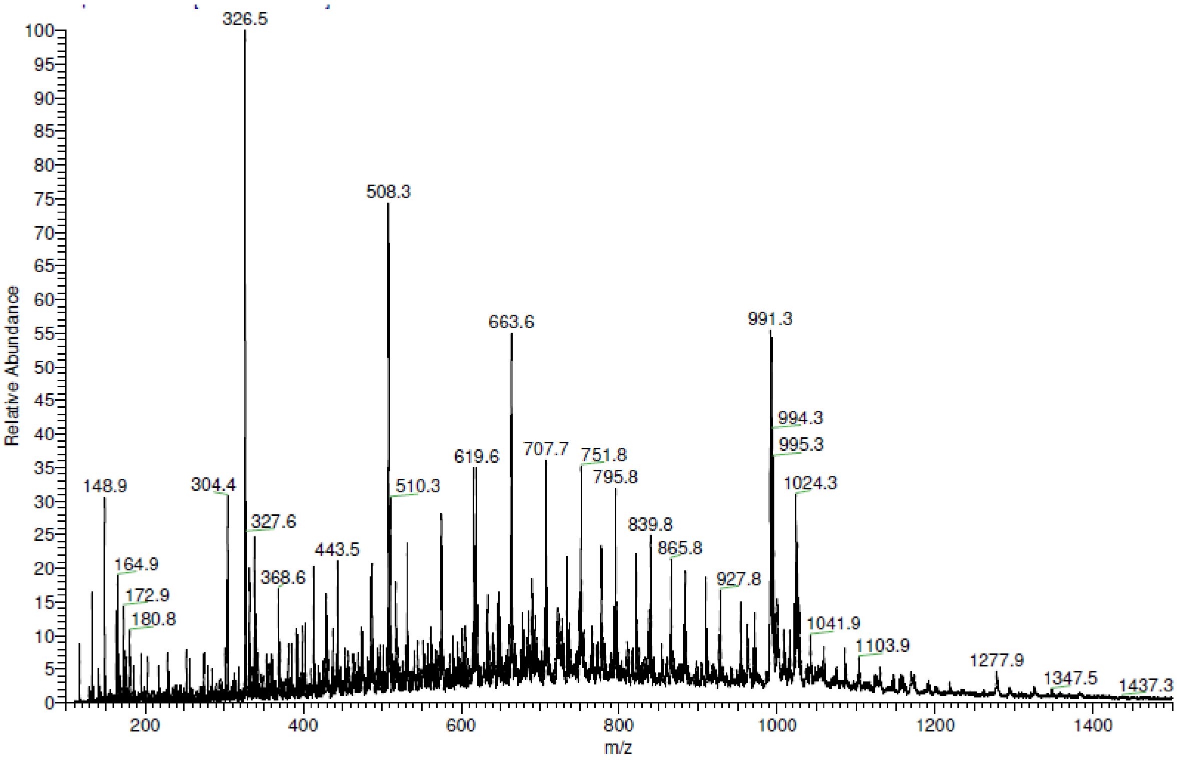

Figure S44. MS-ESI(+) spectrum of **5c**.

Differential Pulse Voltammetry Results

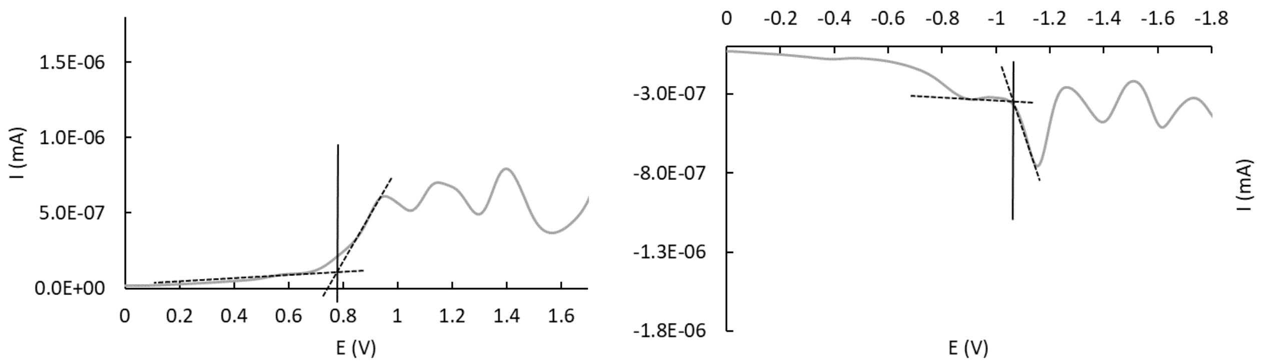

Figure S45. Positive (left) and negative (right) DPV (E vs. SCE) voltammograms of compound **5a** (solution of 0.5 mM with 0.1 M $TBAPF_6$ in CH_2Cl_2) at scan rate of 20 mV s^{-1}.

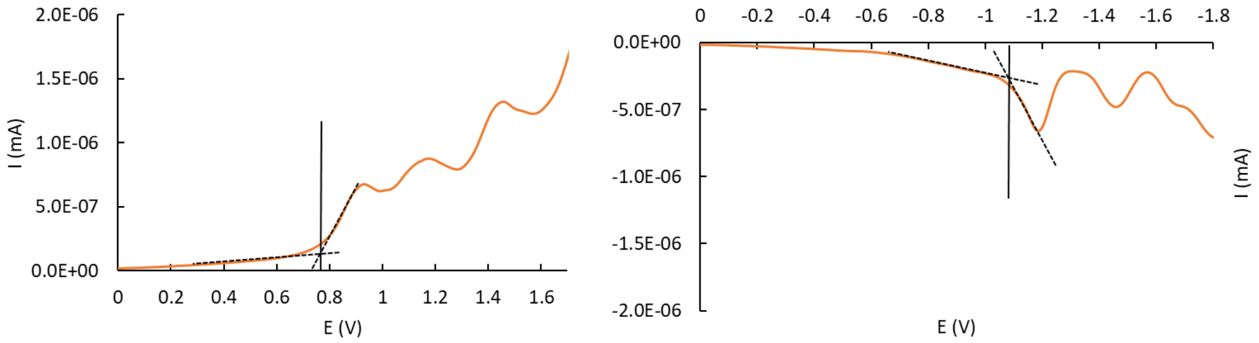

Figure S46. Positive (left) and negative (right) DPV (E vs. SCE) voltammograms of compound **5b** (solution of 0.5 mM with 0.1 M $TBAPF_6$ in CH_2Cl_2) at scan rate of 20 mV s^{-1}.

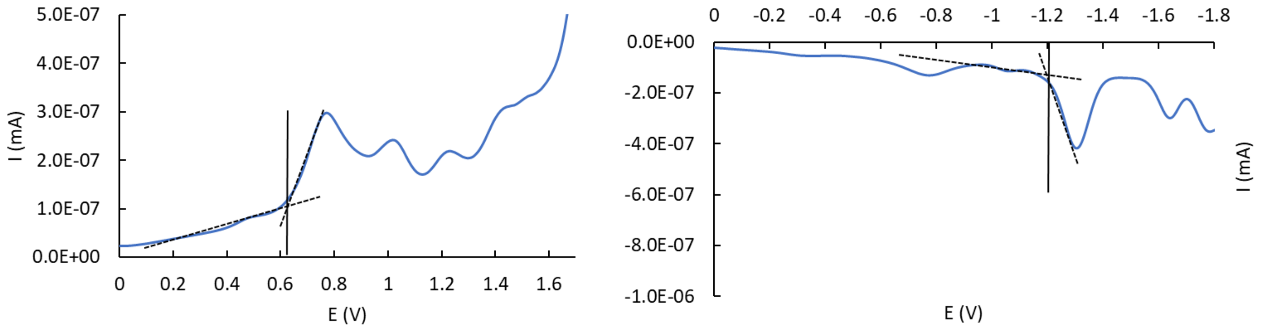

Figure S47. Positive (left) and negative (right) DPV (E vs. SCE) voltammograms of compound **5c** (solution of 0.5 mM with 0.1 M $TBAPF_6$ in CH_2Cl_2) at scan rate of 20 mV s^{-1}.

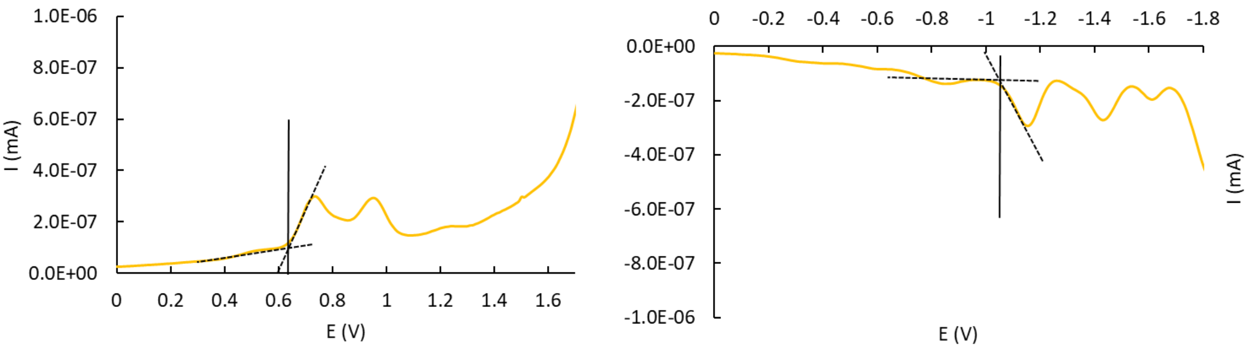

Figure S48. Positive (left) and negative (right) DPV (E vs. SCE) voltammograms of compound **6a** (solution of 0.5 mM with 0.1 M $TBAPF_6$ in CH_2Cl_2) at scan rate of 20 mV s^{-1}.

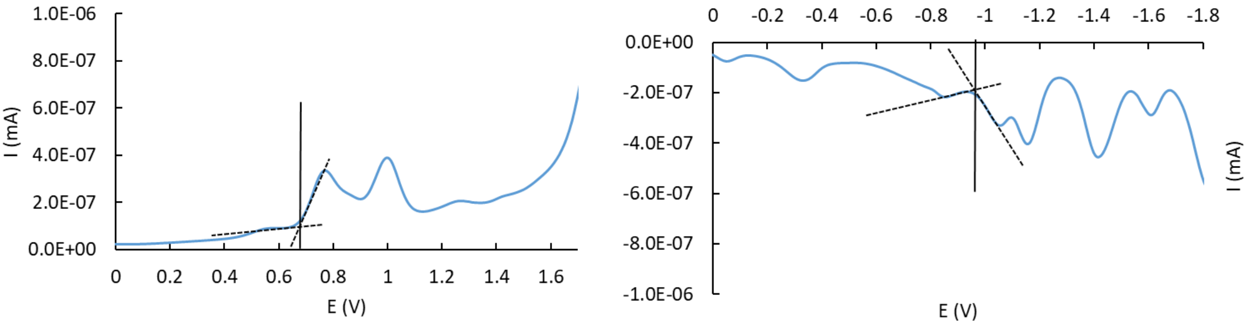

Figure S49. Positive (left) and negative (right) DPV (E vs. SCE) voltammograms of compound **6b** (solution of 0.5 mM with 0.1 M $TBAPF_6$ in CH_2Cl_2) at scan rate of 20 mV s^{-1}.
